# SELL Marks an Effector‐Deficient CD8^+^ T Cell Subset That Promotes Intracerebral Hemorrhage and Responds to Rutin Therapy

**DOI:** 10.1155/humu/4033506

**Published:** 2026-04-02

**Authors:** Yan Huang, Haochen Xu, Congxia Bai, Jing Liu, Yingying Sun, Jingzhou Chen

**Affiliations:** ^1^ State Key Laboratory of Cardiovascular Disease, Fuwai Hospital, National Center for Cardiovascular Diseases, Chinese Academy of Medical Sciences and Peking Union Medical College, Beijing, China, cacms.ac.cn; ^2^ Department of Clinical Laboratory Medicine, Xijing Hospital, Fourth Military Medical University, Xi′an, China, fmmu.edu.cn; ^3^ National Health Commission Key Laboratory of Cardiovascular Regenerative Medicine, Fuwai Central-China Hospital, Central-China Branch of the National Center for Cardiovascular Diseases, Zhengzhou, China; ^4^ Institute of Cardiovascular Disease, Henan Academy of Innovations in Medical Science, Zhengzhou, China

**Keywords:** biomarker, intracerebral hemorrhage, machine learning, rutin, SELL/CD62L, single-cell RNA sequencing, T cells

## Abstract

**Background:**

Intracerebral hemorrhage (ICH) is a devastating stroke subtype with high mortality and limited therapeutic options. While neuroinflammation contributes to secondary brain injury, the role of peripheral CD8^+^ T cell dysfunction in ICH pathogenesis remains poorly characterized. This study is aimed at identifying disease‐associated CD8^+^ T cell subpopulations and potential therapeutic targets through integrative multiomics analysis.

**Methods:**

We performed bulk RNA sequencing on peripheral blood from 130 patients (66 ICH and 64 hypertension controls) across two independent cohorts, combined with single‐cell RNA sequencing of 13 patients. The scPAS algorithm integrated bulk and single‐cell data to identify phenotype‐associated cells. Five machine learning algorithms (LASSO, random forest, XGBoost, SVM, and Boruta) were employed for biomarker discovery. The therapeutic efficacy of rutin was evaluated in murine hypertensive ICH models.

**Results:**

We identified a distinct SELL‐high CD8^+^ T cell subpopulation (scPAS^+^ cells) exhibiting comprehensive effector dysfunction, characterized by downregulation of cytotoxicity genes (GZMA, GZMB, GNLY, NKG7, and CCL5). Pseudotime trajectory analysis revealed progressive differentiation toward this dysfunctional phenotype. SELL emerged as a consensus diagnostic biomarker across all five algorithms, demonstrating excellent discriminative performance (AUC: 0.876–0.936). In vivo, rutin treatment reduced SELL expression, restored CD8^+^ T cell cytotoxicity, decreased hemorrhage incidence, and attenuated neuroinflammation and oxidative stress.

**Conclusions:**

This study identifies SELL‐marked effector‐deficient CD8^+^ T cells as a hallmark of ICH and establishes SELL as a robust diagnostic biomarker. Rutin represents a promising therapeutic candidate targeting peripheral immune dysfunction in hypertensive ICH.

## 1. Introduction

Intracerebral hemorrhage (ICH) represents a catastrophic cerebrovascular event characterized by spontaneous bleeding within the brain parenchyma, accounting for approximately 10%–15% of all stroke cases worldwide [1, 2]. Despite constituting a minority of stroke subtypes, ICH disproportionately contributes to stroke‐related mortality and long‐term disability, with case fatality rates approaching 50% within the first month following onset [3]. Recent epidemiological analyses utilizing data from the Global Burden of Disease 2021 study have documented approximately 3.44 million new ICH cases annually, underscoring the substantial public health implications of this condition [4]. The disease burden is particularly pronounced in low‐ and middle‐income countries, where inadequate management of modifiable risk factors perpetuates elevated incidence rates [5]. Chronic arterial hypertension (HTN) constitutes the predominant etiological factor underlying spontaneous ICH, inducing progressive pathological alterations in cerebral microvasculature, including arteriolar hyalinosis, lipohyalinosis, and fibrinoid necrosis [6, 7]. These degenerative processes compromise vascular integrity, predisposing penetrating arteries to rupture upon acute hemodynamic stress. The resultant parenchymal hemorrhage initiates a cascade of secondary injury mechanisms encompassing perihematomal edema formation, oxidative stress propagation, and neuroinflammatory amplification, collectively exacerbating neurological deterioration [8, 9].

Accumulating evidence has positioned the immune system as a central orchestrator of posthemorrhagic brain injury. The inflammatory response following ICH involves coordinated interactions between brain‐resident immune cells and peripherally derived leukocytes that infiltrate the hemorrhagic lesion [10, 11]. Among circulating immune populations, T lymphocytes have emerged as particularly consequential mediators of secondary brain damage. Flow cytometric analyses have demonstrated temporal increases in brain‐infiltrating CD4^+^ T cells during the acute phase of experimental ICH, with their presence correlating with blood–brain barrier disruption and edema expansion [12, 13]. Notably, CD8^+^ cytotoxic T lymphocytes have been implicated in neuronal apoptosis induction and exacerbation of immune‐mediated tissue damage in cerebrovascular pathologies [14, 15].

Single‐cell RNA sequencing (scRNA‐seq) technologies have revolutionized our capacity to delineate cellular heterogeneity within complex immunological landscapes at unprecedented resolution [16, 17]. This methodological advancement enables unbiased identification of discrete cell subpopulations and their transcriptional signatures, thereby illuminating previously obscured aspects of disease pathophysiology. Application of scRNA‐seq to stroke research has yielded transformative insights into the phenotypic diversity of infiltrating immune cells and their dynamic trajectory transitions during disease progression [18, 19]. However, systematic investigation of peripheral blood CD8^+^ T cell alterations in ICH patients utilizing single‐cell approaches remains limited.

SELL functions as a critical adhesion molecule governing lymphocyte trafficking and homing to secondary lymphoid organs [20, 21]. Beyond its canonical role in cellular migration, emerging evidence suggests that SELL participates in modulating T cell effector functions. Proteolytic shedding of membrane‐bound SELL accompanies T cell activation and correlates with acquisition of cytotoxic capacity [22]. Furthermore, SELL expression serves to distinguish central memory T cells (SELL^+^) from effector memory populations (SELL^−^), with the former demonstrating superior proliferative potential and longevity [23, 24]. The functional significance of SELL dysregulation in ICH pathogenesis, however, remains inadequately characterized.

Rutin (quercetin‐3‐O‐rutinoside) represents a naturally occurring flavonoid glycoside abundantly distributed in various botanical sources, including buckwheat, citrus fruits, and *Sophora japonica* [25, 26]. Extensive pharmacological investigations have established rutin′s multifaceted bioactivities encompassing antioxidant, anti‐inflammatory, and cytoprotective properties [27]. Notably, rutin has demonstrated neuroprotective efficacy in experimental models of cerebral ischemia through attenuation of oxidative stress, suppression of matrix metalloproteinase activity, and preservation of blood–brain barrier integrity [28, 29]. Recent studies employing zebrafish models have further documented rutin′s capacity to ameliorate hemorrhagic stroke via modulation of the GSK‐3*β*/Nrf2 signaling axis [30]. Nevertheless, the therapeutic potential of rutin in targeting specific immune cell populations during ICH progression warrants systematic exploration.

To leverage the robust clinical signals from large‐scale bulk transcriptomes and the cellular granularity of scRNA‐seq, we employed the scPAS framework—a validated phenotype‐projection methodology [31, 32]. By utilizing disease‐specific signatures from bulk cohorts to identify phenotype‐associated cell states, this approach bridges the gap between population‐level clinical relevance and single‐cell resolution. This strategy ensures that the identified immune cell alterations are directly linked to the ICH phenotype, overcoming the common limitations of small sample sizes and lack of clinical representativeness in traditional single‐cell studies.

In the present investigation, we integrated multiomics transcriptomic profiling with advanced machine learning algorithms to comprehensively characterize peripheral blood CD8^+^ T cell dysfunction in ICH patients. Through convergent analysis of bulk and single‐cell transcriptomic datasets, we identified an effector‐deficient CD8^+^ T cell subset marked by elevated SELL expression that associates with ICH pathogenesis. Furthermore, we demonstrated that pharmacological targeting of SELL using rutin confers protection against hypertensive cerebral hemorrhage in murine models. These findings provide novel mechanistic insights into peripheral immune dysregulation during ICH and identify actionable therapeutic targets for this devastating cerebrovascular condition.

## 2. Materials and Methods

### 2.1. Enrollment of Patients

This investigation adhered to the principles outlined in the Declaration of Helsinki and received institutional review board approval from Fuwai Hospital (Protocol No. 2024‐2458). Prior to participation, all subjects provided documented informed consent. For comprehensive characterization of immunological alterations and the mechanistic underpinnings of ICH, two separate patient populations were assembled. Between 2014 and 2019, a total of 66 individuals presenting with clinical features consistent with ICH were recruited alongside 64 demographically matched hypertensive controls for transcriptomic profiling. The initial cohort, assembled at Cangzhou Central Hospital during 2014–2017, consisted of 46 ICH cases paired with 46 hypertensive subjects. The subsequent validation cohort incorporated 20 ICH patients from Hebei University Affiliated Hospital and 18 hypertensive individuals from Ningxia Medical University General Hospital. Eligibility for the hypertensive control arm required documented HTN without antecedent cerebrovascular or cardiovascular events. ICH diagnosis was confirmed through neuroimaging, with exclusion of alternative hemorrhagic etiologies. Comprehensive clinical evaluation encompassing standardized interviews and medical record review enabled systematic exclusion of concomitant autoimmune disorders, cardiovascular conditions, hepatorenal dysfunction, and malignancies. To minimize the variability associated with the dynamic immune response following ICH, peripheral blood samples were collected using a standardized temporal protocol. All samples were obtained within 24 h of patient admission.

### 2.2. Animal Treatment

Animal experimentation protocols received approval from the Institutional Animal Care Committee at Fuwai Hospital and were executed following ARRIVE reporting standards and applicable national regulations. To eliminate sex‐based hormonal variability, exclusively male C57BL/6N mice aged 8 months (body weight: 28–34 g) were obtained from the National Resource Center of Model Mice. Animals underwent environmental adaptation within specific pathogen‐free facilities maintained under controlled conditions (alternating 12‐h photoperiods and unrestricted food and water access). Randomization to experimental arms was accomplished using SPSS‐generated allocation sequences following anesthetic induction. Blinded assessment protocols were implemented throughout data collection and subsequent analyses to minimize observer bias.

### 2.3. Bulk Transcriptome Sequencing

Peripheral blood specimens underwent total RNA extraction, with library preparation and sequencing services provided by Annoroad Gene Technology (Beijing, China). Quality metrics, including RNA integrity and purity, were evaluated via Labchip GX Touch capillary electrophoresis and Nanodrop 2000 spectrophotometry, respectively. Library construction proceeded through mRNA enrichment using oligo(dT)‐conjugated magnetic beads, followed by fragmentation and reverse transcription to generate double‐stranded cDNA. Standard library preparation workflows included end‐repair, adenylation, adapter ligation, size selection targeting approximately 350 bp inserts, and PCR enrichment. Presequencing quality verification encompassed concentration measurement (Qubit 3.0 fluorometry) and insert size profiling (Agilent 2100 Bioanalyzer). Bioinformatic processing initiated with quality filtration to eliminate adapter contamination, low‐quality base calls, and sequences containing excessive undefined nucleotides. Cleaned reads underwent alignment to the ENSEMBL human reference assembly via HISAT2, with transcript abundance quantification expressed as TPM‐normalized values. Identification of differentially expressed transcripts employed DESeq2′s negative binomial modeling framework, applying Benjamini–Hochberg correction with significance defined at FDR < 0.05. Pathway enrichment analyses interrogating Gene Ontology (GO) and KEGG databases were conducted through clusterProfiler (significance: *p* < 0.05).

### 2.4. Human Single‐Cell Sequencing

scRNA‐seq specimens were obtained from the First Affiliated Hospital of Dalian Medical University, comprising eight cerebral microbleed (MB) patients and six age‐ and sex‐matched hypertensive controls. Inclusion criteria mandated untreated Grade 3 HTN (systolic pressure ≥ 160 mmHg and diastolic pressure ≥ 100 mmHg). MB diagnosis required neurologist confirmation with radiological documentation of three or more characteristic lesions (2–5 mm diameter, hypointense signal, and spherical/ovoid morphology) localized to basal ganglia structures. Rigorous exclusion parameters eliminated individuals with concurrent neurological pathology, systemic inflammatory conditions, or—specifically for controls—any cerebrovascular history. Demographic and clinical parameters were ascertained through direct patient interviews complemented by a comprehensive medical documentation review. Peripheral blood samples were strictly collected from all patients within 24–48 h after the confirmed onset of hypertensive ICH symptoms, ensuring a consistent window to capture the peak acute‐phase immune response while minimizing potential confounding from late‐stage systemic complications. Ethical oversight was provided by the Fuwai Hospital Human Research Ethics Committee (Protocol No. 2022‐1692), with adherence to Good Clinical Practice standards and Helsinki Declaration principles. All participants or designated representatives executed written consent documentation.

Transcriptomic profiling at single‐cell resolution utilized the BD Rhapsody platform. Cellular suspensions were partitioned into nanowell arrays through limiting dilution, with subsequent bead loading achieving single‐cell‐bead colocalization per microwell. Cell lysis enabled mRNA capture onto barcoded beads, followed by reverse transcription, generating cDNA molecules incorporating cell‐specific barcodes and unique molecular identifiers. Transcriptome‐wide amplification proceeded according to manufacturer specifications. Quantification of amplified libraries (Agilent Bioanalyzer; Qubit HS DNA assay) preceded sequencing on Illumina instrumentation using 150 bp paired‐end chemistry.

### 2.5. Single‐Cell Data Processing Pipeline

Quality control followed established protocols within the R computing environment. The Seurat package (v4.0.4) Read10X function imported raw count matrices into sparse dgCMatrix format. Dataset integration created unified Seurat objects with RenameCells, assigning distinct cellular identifiers. The DoubletFinder algorithm eliminated putative doublets. Filtering removed cells expressing fewer than 200 genes and genes present in fewer than three cells [33]. Samples yielding insufficient high‐quality cells (< 1000) were excluded to maintain adequate representation. LogNormalize transformation (scale factor: 10,000) preceded identification of 2000 highly variable features via FindVariableFeatures. Technical confounders, including total UMI burden and mitochondrial transcript proportion, were regressed using ScaleData. Principal component analysis on variable genes retained 30 components for downstream processing. The Harmony algorithm corrected intersample batch variation. UMAP embedding visualized the cellular landscape, while FindNeighbors constructed shared nearest‐neighbor graphs, and FindClusters implemented Louvain‐based clustering [34]. Resolution parameters spanning 0.1–2 were evaluated to achieve stable subpopulation delineation. Marker gene identification through FindAllMarkers, combined with literature‐curated references and the Cell Taxonomy repository (https://ngdc.cncb.ac.cn/celltaxonomy/), enabled cell type annotation.

### 2.6. Intercellular Communication Inference

The CellChat framework (v2.1.2) inferred cell–cell interaction networks using its curated ligand–receptor database (http://www.cellchat.org/) [35]. For each cellular population, expressed ligands, receptors, and cofactors were mapped to reference signaling cascades to assess communication potential. Expression‐based probability estimation quantified interaction strength, with netVisual_bubble generating global signaling visualizations using default settings.

### 2.7. Computational Docking Analysis

Virtual screening explored binding configurations and affinities between small molecules and SELL. The three‐dimensional protein structure was obtained from AlphaFold (https://alphafold.ebi.ac.uk/entry/AF-P14151-F1). Preprocessing removed nonprotein atoms and added hydrogen atoms for accurate protonation. An FDA‐approved compound library from MCE underwent MM2 force field energy minimization, with Open Babel (v3.1.1) converting structures to pdbqt format for AutoDock Vina (v1.2.3) compatibility. Absent experimentally validated binding pockets necessitated blind docking with search grids encompassing the entire protein surface. Vina‐predicted binding energies estimated ligand affinity, where lower values indicated stronger interactions. Top‐scoring complexes underwent structural visualization and interaction analysis in PyMOL (v2.3).

### 2.8. Establishment of HTN and Hypertensive ICH Models

This experimental strategy included four mouse groups. In Group 1, a hypertensive ICH model was established by subcutaneous implantation of osmotic minipumps for 28 days (Ang II infusion rate: 490 ng/kg/min), while mice were provided ad libitum access to drinking water containing L‐NAME (100 mg/kg). On Day 7 after pump implantation, Ang II was administered subcutaneously at a dose of 0.5 mg/kg at least twice daily. Systolic arterial blood pressure was measured on Day 5 after model induction to confirm successful HTN. In Group 2, a hypertensive mouse model was generated by subcutaneous implantation of osmotic minipumps combined with daily subcutaneous injections of Ang II (0.5 mg/kg). Mice were closely monitored, and once clinical signs consistent with hypertensive ICH were observed, peripheral blood samples were immediately collected for flow cytometric sorting. Groups 3 and 4 were used to evaluate pharmacological intervention targeting SELL; mice received rutin (100 mg/kg; HY‐N0148, MedChemExpress) or an equivalent volume of DMSO as a vehicle control via daily intraperitoneal injection. This dosage was selected based on prior studies demonstrating its neuroprotective efficacy in rodent models of acute brain injury [36, 37]. Treatment was initiated 2 weeks prior to the onset of ICH and continued until the appearance of stroke symptoms, at which time brain tissues were harvested for subsequent molecular analyses.

### 2.9. Magnetic Resonance Imaging and Volumetric Analysis of ICH

Cerebral hemorrhage was assessed in vivo using a 7 T horizontal‐bore MRI system (Varian). Mice were anesthetized with 2% isoflurane delivered in a 1:1 O_2_/medical air mixture (1 L/min total flow) and maintained under anesthesia throughout the imaging procedure. A T2∗‐weighted gradient‐echo sequence was employed for hemorrhage detection with the following parameters: repetition time = 250 ms, echo time = 5 ms, field of view = 20 × 20 mm, and matrix size = 256 × 256. Twelve consecutive coronal slices (0.5 mm) covering the brain from the frontal pole to the brainstem were acquired. Images were saved in DICOM format. For volumetric analysis, the hyperintense hemorrhagic lesion on each T2∗‐weighted image was manually outlined by an investigator blinded to the experimental groups using ImageJ software. Total hemorrhage volume was calculated by summing the traced areas across all consecutive slices and multiplying by the slice thickness.

### 2.10. Flow Cytometric Cell Sorting

Peripheral blood leukocytes were isolated from 1 mL heparinized specimens through sequential erythrocyte depletion using 10 mL 1× hypotonic lysis buffer (two cycles: gentle mixing, 5‐min ice incubation, centrifugation at 400 × *g* for 5 min at 4°C). Recovered cells underwent dual PBS/HBSS washes followed by equilibration in protein‐free, Tris‐free PBS. Viability assessment employed Zombie Aqua fixable dye (1:1000 dilution; BioLegend #423101) with 15‐min ambient temperature incubation protected from light. Postwash surface epitope labeling utilized predetermined antibody panels (30 min, 4°C, light‐protected). Final preparations were filtered through 300‐mesh strainers to ensure single‐cell dispersions suitable for high‐speed sorting on a BD FACSAria II instrument. Data interpretation utilized FlowJo v10.7 analytical software. The antibody panel comprised the following: CD45‐specific reagent (BioLegend #103130), CD3‐targeting antibody (BioLegend #100205), CD8 detection reagent (BioLegend #A15386), and CD62L‐directed antibody (BioLegend #104411).

### 2.11. Enzyme‐Linked Immunosorbent Assays

Circulating concentrations of SELL, TNF‐*α*, and IL‐6 were determined using commercially available sandwich ELISA platforms (Elabscience). Assay components were temperature‐equilibrated for 20 min prior to use. Standards and test specimens (100 *μ*L volumes) were dispensed into antibody‐precoated microplate wells and incubated at 37°C for 90 min. Detection proceeded through sequential additions of biotinylated secondary antibody (37°C, 60 min) and streptavidin–HRP conjugate (37°C, 30 min), with intervening wash cycles (three and five repetitions, respectively). Chromogenic development utilized 90 *μ*L tetramethylbenzidine substrate (37°C, 15 min), with reaction termination via 50 *μ*L acidic stop solution. Optical density measurements at 450 nm enabled concentration determination through standard curve interpolation using ELISACalc software.

### 2.12. Immunofluorescent Staining

Cryosectioned tissues underwent immunolabeling following established institutional protocols. Primary antibody reagents included the following: rabbit anti‐CD8a (Abcam ab316778), rat anti‐CD62L/SELL (Abcam ab119834), Cy3‐conjugated *α*‐SMA antibody (Sigma‐Aldrich C6198, clone 1A4), goat anti‐Iba‐1 (Abcam ab5076), and goat anti‐CD31 (R&D Systems AF3628). Secondary detection employed Alexa Fluor–conjugated donkey immunoglobulins: antigoat IgG‐594, antirat IgG‐594, antirat IgG‐488, antirabbit IgG‐488, and antimouse IgG‐488 (Abcam). Image acquisition utilized Leica SP8 confocal and DM6000B widefield microscopy systems. Microglial activation status was assigned based on combined morphological assessment and Iba‐1‐positive soma diameter thresholds (< 7.5 *μ*m indicating resting phenotype). Type IV collagen coverage was calculated as a percentage area overlapping CD31‐positive endothelium within 0.078 mm^2^ sampling regions. Quantitative analyses sampled four randomly selected fields per section (anteroposterior coordinates: 1.2, 2.2, and 3.2 mm relative to bregma) across three tissue sections using 40× magnification.

### 2.13. TUNEL Staining

Programmed cell death was visualized through terminal deoxynucleotidyl transferase‐mediated dUTP nick‐end labeling. Cryosections underwent paraformaldehyde fixation (4% concentration) and membrane permeabilization (0.1% Triton X‐100 in 0.1% sodium citrate solution). Apoptotic nuclei were labeled using the In Situ Cell Death Detection system (Roche #11684795910) per manufacturer guidelines, involving TdT‐mediated incorporation of fluorescent nucleotides at DNA strand breaks. Nuclear counterstaining employed DAPI. Negative control sections omitted the TdT enzyme from the reaction mixtures. Confocal microscopy enabled image capture, with apoptotic indices calculated as TUNEL^+^/DAPI^+^ dual‐positive cells enumerated across four randomized fields per section, three sections per specimen (*n* = 6 biological replicates per experimental arm).

### 2.14. Quantitative Real‐Time PCR

RNA was purified from flow‐sorted CD8^+^ T cells using RNeasy Micro columns (QIAGEN). First‐strand cDNA synthesis employed PrimeScript RT reagents (TaKaRa). Quantitative amplification was performed on a CFX96 thermal cycler (Bio‐Rad) with ChamQ SYBR Green chemistry (Vazyme #Q311‐02). Relative transcript abundance was computed via the comparative threshold cycle approach (2^−*Δ* *Δ*Ct^), with GAPDH serving as normalization reference.

Murine primer sequences employed were as follows:

GAPDH: 5 ^′^‐CACATGGCCTCCAAGGAGTAA‐3 ^′^ (sense); 5 ^′^‐TGAGGGTCTCTCTCTTCCTCTTGT‐3 ^′^ (antisense)

SELL: 5 ^′^‐AACGAGACTCTGGGAAGT‐3 ^′^ (sense); 5 ^′^‐CAAAGGCTCACATTGGAT‐3 ^′^ (antisense)

GZMA: 5 ^′^‐AGGTGGAAGAGACTCGTGCAA‐3 ^′^ (sense); 5 ^′^‐GTCTCCGCATTTATTTTCAAG‐3 ^′^ (antisense)

GZMB: 5 ^′^‐TGCGAATCTGACTTACGCCAT‐3 ^′^ (sense); 5 ^′^‐GGAGGCATGCCATTGTTTCG‐3 ^′^ (antisense)

CCL5: 5 ^′^‐CCTGCTGCTTTGCCTACCTCTC‐3 ^′^ (sense); 5 ^′^‐ACACACTTGGCGGTTCCTTCGA‐3 ^′^ (antisense)

NKG7: 5 ^′^‐CCACAGGTCCTCACTTCTCTGC‐3 ^′^ (sense); 5 ^′^‐CAGCCAGGATACAGAAGCTCTG‐3 ^′^ (antisense)

GNLY: 5 ^′^‐GAAGAAGATGGTGGATAAG‐3 ^′^ (sense); 5 ^′^‐CTAGACTGATACCTCCTC‐3 ^′^ (antisense)

GZMH: 5 ^′^‐CTGCTCACTGTGAAGGAAGTATAA‐3 ^′^ (sense); 5 ^′^‐TCAGCTCTAGGGACGATGGG‐3 ^′^ (antisense)

### 2.15. Western Blot

Fluorescence‐activated cell sorting (FACS)–purified CD8^+^ T cells were solubilized in RIPA extraction buffer containing protease/phosphatase inhibitor cocktails (Roche). Following 30‐min ice incubation, lysates were clarified by centrifugation (16,000 × *g*, 15 min, 4°C). Total protein quantification utilized bicinchoninic acid colorimetric assay (Pierce). Equivalent protein masses were heat‐denatured in reducing Laemmli buffer (5% *β*‐mercaptoethanol), resolved on 4%–20% gradient SDS–polyacrylamide gels (Bio‐Rad), and electrotransferred to PVDF membranes (Millipore) via semidry blotting. Immunodetection employed primary antibodies targeting CD62L (Cell Signaling Technology #45658; 1:2000 dilution) and GAPDH loading control (Proteintech #60004‐1‐Ig; 1:2000 dilution).

### 2.16. Tissue Processing and Hemorrhage Analysis

Extracted brains underwent overnight immersion fixation in 4% paraformaldehyde (4°C), sequential cryoprotection in ascending sucrose gradients (20% and 30%), and coronal cryosectioning at 20 *μ*m intervals. Histomorphological evaluation employed hematoxylin–eosin chromatic staining of every fifth serial section. Brightfield photomicrographs were obtained using Leica DM6000B optics. Hemorrhagic lesion areas were delineated by treatment‐blinded observers using ImageJ morphometry. Complementary biochemical hemorrhage quantification involved homogenization of ipsilateral hemispheric tissue followed by spectrophotometric hemoglobin determination (Drabkin′s reagent; Sigma‐Aldrich) at 540 nm absorbance, referenced against standard concentration curves.

### 2.17. Cerebral Oxidative Stress Evaluation

Superoxide anion production within neural tissue was assessed through dihydroethidium (DHE) fluoroprobe oxidation. Freshly excised brain specimens were OCT‐embedded and rapidly cryopreserved. Coronal cryosections (10 *μ*m thickness) were incubated with DHE (4 *μ*M in Krebs–Henseleit solution; 37°C, 30 min, light‐protected). Postincubation washing preceded confocal fluorescence imaging (Leica; 40× objective). Superoxide‐dependent ethidium fluorescence (red channel) was quantified as mean intensity values across defined regions of interest in a minimum of six sections per animal, with automated background subtraction performed in ImageJ.

### 2.18. Identifying High‐Risk Cell Subpopulations Linked to Bulk‐Level Phenotypes via Single‐Cell Transcriptomics

To delineate cell subpopulations associated with bulk‐defined clinical phenotypes, we applied scPAS, a computational framework that bridges bulk transcriptomic profiles with scRNA‐seq data. This approach enables the prioritization of cells exhibiting strong transcriptional associations with distinct phenotypic states [31, 32]. In the present study, specimens derived from ICH and HTN patients were assigned binary labels (1 and 0, respectively) to facilitate supervised classification. The analysis was conducted using an imputed gene expression matrix to mitigate the impact of dropout events inherent to scRNA‐seq data. To constrain the proportion of scPAS^+^ and scPAS^−^ cells within 20% of the overall cell population, the alpha parameter was calibrated to 0.1. Underlying the framework is a logistic regression model that assigns per‐gene coefficient (coef) values, which collectively support phenotype prediction as well as cross‐cohort transferability for inference on independent external datasets.

### 2.19. Statistical Methods

Parametric continuous outcomes satisfying normality assumptions were compared using unpaired Student′s *t*‐tests; nonparametric alternatives (Mann–Whitney *U*) were applied otherwise. Categorical variable associations were evaluated through chi‐square testing or Fisher′s exact probability calculations based on expected frequency distributions. Bidirectional hypothesis testing was conducted throughout using the R statistical computing environment, with significance established at *α* = 0.05.

## 3. Result

### 3.1. Multiomics Integrative Analysis Decodes the Critical Role of CD8^+^ T Cells in the Peripheral Blood of ICH Patients

We collected peripheral blood samples from patients with HTN and ICH across two independent cohorts and performed bulk RNA sequencing (Figure 1a). Cohort 1 comprised 46 HTN and 46 ICH samples, while Cohort 2 included 18 HTN and 20 ICH samples. Given its larger sample size, Cohort 1 served as the discovery cohort for primary analysis, with Cohort 2 designated as an independent validation cohort for subsequent verification. To maintain consistency and allow for direct comparison, we adhered to the standardized data processing pipeline detailed in our earlier study [38]. Gene Set Enrichment Analysis (GSEA) revealed significant suppression of multiple T cell function–related pathways, including the T cell receptor signaling pathway, positive regulation of the T cell receptor signaling pathway, alpha–beta T cell activation, and T cell activation (Figure 1b). However, bulk transcriptomics inherently lacks the resolution to directly delineate T cell–specific alterations in ICH due to technical limitations. The advent of scRNA‐seq technology provides an unprecedented opportunity to investigate transcriptional changes in ICH at single‐cell resolution.

Figure 1Multiomics integrative analysis decodes the critical role of CD8^+^ T cells in the peripheral blood of ICH patients. (a) Schematic of bulk RNA‐seq data collection and sequencing workflow. (b) GSEA results of ICH patients. (c) Schematic of single‐cell RNA sequencing sample collection and workflow. (d) UMAP visualization of cells from each sample. (e) UMAP visualization after cell type annotation. (f) Bubble plot showing the expression of canonical marker genes across cell types. (g) Annotation of CD4^+^ T and CD8^+^ T cell subsets. (h) Quantification of CD4^+^ T and CD8^+^ T cell contributions to the ICH phenotype using the scDist algorithm. (i) Identification of phenotype‐associated cells using scPAS analysis. UMAP visualization of the NRS distribution, where the color gradient represents the strength of phenotype association (left). Stratification of cells into three states based on NRS: scPAS^+^ (ICH‐associated, red), scPAS^−^ (HTN‐associated, blue), and mixed (intermediate cells, grey) (right). (j) Coefficient values of top genes in the logistic regression model fitted by the scPAS algorithm. (k) Validation of the logistic regression model in bulk transcriptomic data.

**Figure:**
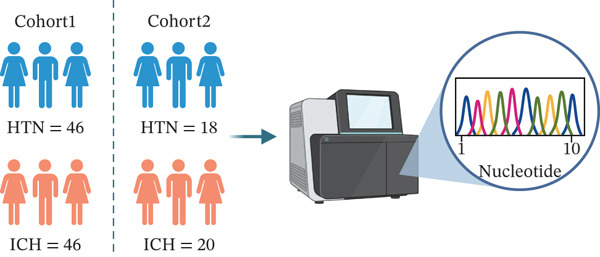
(a)

**Figure:**
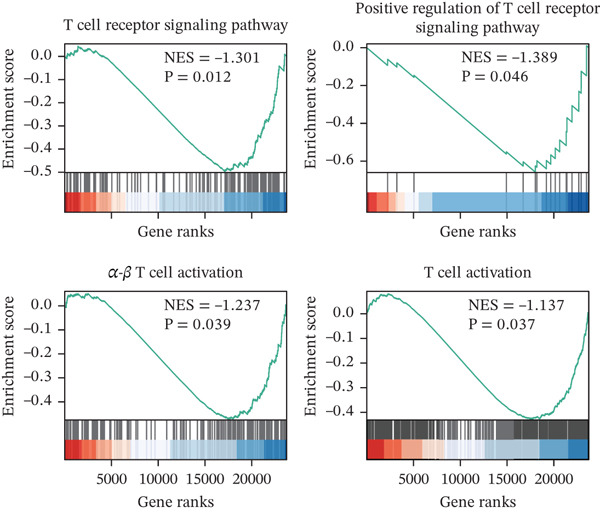
(b)

**Figure:**
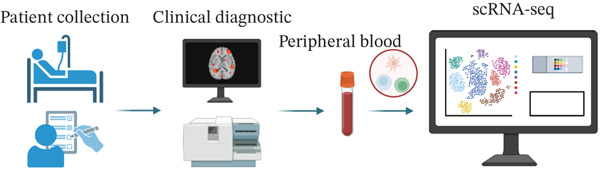
(c)

**Figure:**
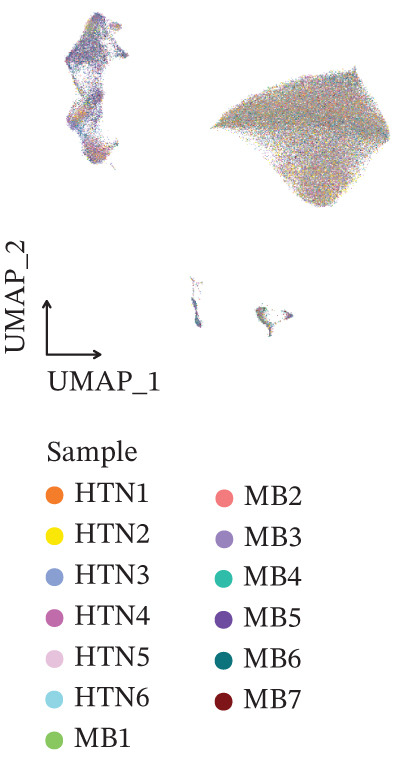
(d)

**Figure:**
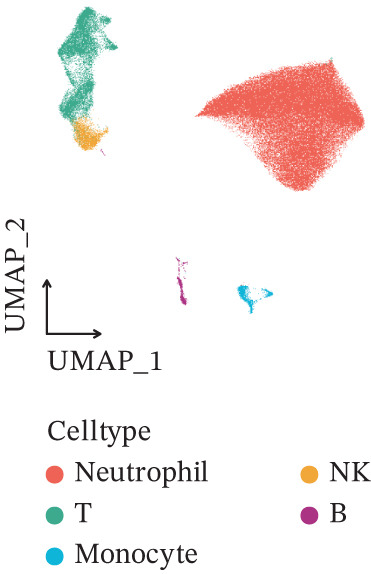
(e)

**Figure:**
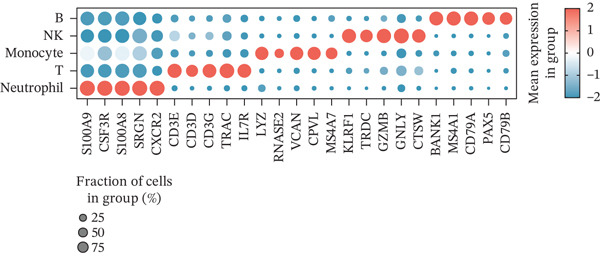
(f)

**Figure:**
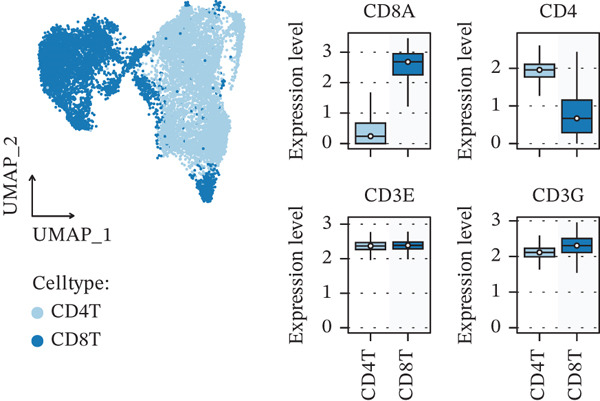
(g)

**Figure:**
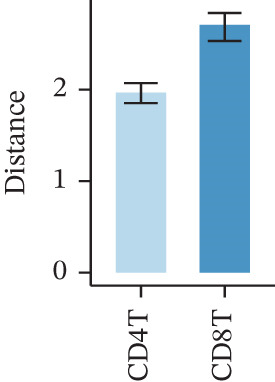
(h)

**Figure:**
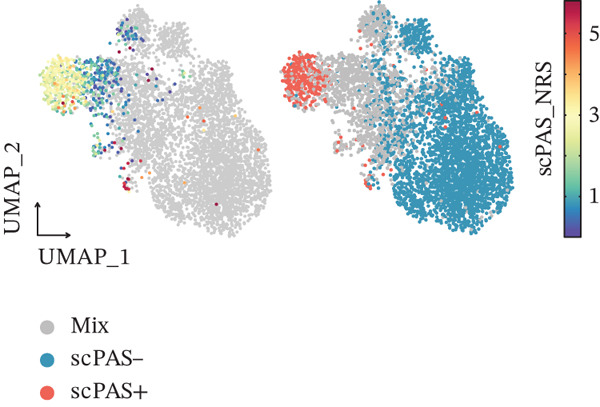
(i)

**Figure:**
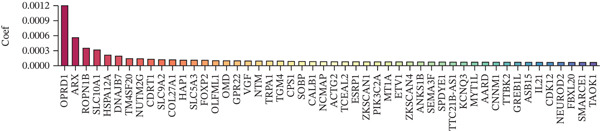
(j)

**Figure:**
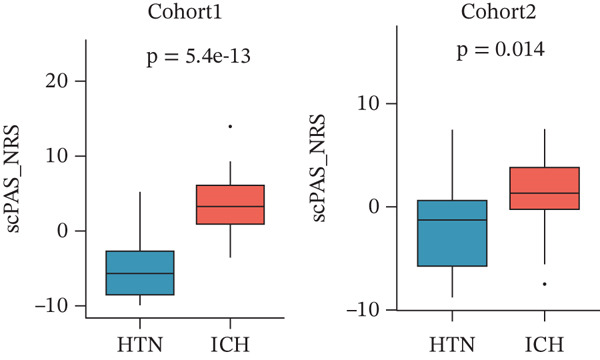
(k)

We performed scRNA‐seq on peripheral blood samples from 13 patients (Figure 1c), comprising six HTN controls and seven patients with cerebral MBs (Figure 1d). A total of 19,541 cells passed stringent quality control criteria. Following batch effect correction, all cells were annotated based on canonical marker gene expression profiles (Figure 1e,f), identifying distinct populations including neutrophils, T cells, monocytes, NK cells, and B cells. Subsequently, we extracted all T cells for further analysis and classified them into CD4^+^ and CD8^+^ T cell subsets (Figure 1g). Notably, scDist analysis demonstrated that CD8^+^ T cells exhibited a substantially higher contribution to the ICH phenotype compared to CD4^+^ T cells (Figure 1h), prompting us to focus our subsequent analyses specifically on the CD8^+^ T cell compartment.

To integrate bulk and single‐cell transcriptomic data, we employed the scPAS algorithm [31]. scPAS generates a normalized risk score (NRS) by fitting a logistic regression model. Strikingly, we observed distinct clustering patterns of NRS values in the dimensionality‐reduced landscape (Figure 1i), suggesting the existence of ICH phenotype–specific CD8^+^ T cell subpopulations. Based on NRS distribution, the scPAS algorithm stratified all cells into scPAS^−^ and scPAS^+^ populations (Figure 1i), where scPAS^+^ cells were associated with the ICH phenotype, scPAS^−^ cells corresponded to the HTN phenotype, and intermediate cells were classified as mixed. Figure 1j displays the coefficient values of core genes in this logistic regression model. To further validate the accuracy of the scPAS model, we applied this logistic regression framework to bulk transcriptomic data from both cohorts. Importantly, NRS values were significantly elevated in ICH patients compared to HTN controls in both Cohort 1 and Cohort 2 (Figure 1k). Collectively, by integrating multiomics data from large‐scale transcriptomic cohorts and leveraging the scPAS algorithm, we established a predictive model for ICH patients that enabled the identification of two phenotypically distinct CD8^+^ T cell subpopulations.

### 3.2. CD8^+^ T Cell Dysfunction Is a Hallmark of ICH Patients

We next performed an in‐depth characterization of the molecular differences between scPAS^+^ and scPAS^−^ cells. GSEA revealed that multiple immune‐related pathways were significantly suppressed in scPAS^+^ cells, including natural killer cell activation involved in immune response, regulation of immune effector process, positive regulation of T cell–mediated cytotoxicity, lymphocyte‐mediated immunity, and cell killing (Figure 2a). Conversely, pathways associated with cation reactions, cytoplasmic translation, and regulation of mRNA processing were upregulated. Furthermore, we observed that cytotoxic effector genes, including CCL5, NKG7, GNLY, GZMH, GZMA, and GZMB, were uniformly downregulated in scPAS^+^ cells (Figure 2b). These findings collectively indicate that scPAS^+^ cells exist in a cytotoxically dysfunctional state.

Figure 2CD8^+^ T cell dysfunction is a hallmark of ICH patients. (a) GSEA of scPAS^+^ cells. (b) Differential expression of cytotoxicity‐related genes between scPAS^−^ and scPAS^+^ cells. (c) Pseudotime trajectory analysis using the Vector algorithm. (d) Pseudotime trajectory analysis using the Monocle3 algorithm. (e) Pseudotime trajectory analysis using the Slingshot algorithm. (f) Pseudotime trajectory analysis using the scTour algorithm. (g) Dynamic changes in the expression of cytotoxicity‐related genes along the differentiation trajectory. (h) Cell type–specific transcription factors with elevated activity. (i) UMAP visualization of the activity and RNA expression levels of transcription factors specifically upregulated in scPAS+ cells. (j) Dynamic changes in the activity of transcription factors specifically upregulated in scPAS+ cells along the differentiation trajectory.

**Figure:**
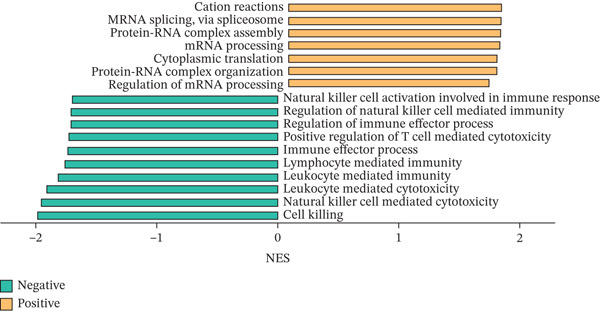
(a)

**Figure:**
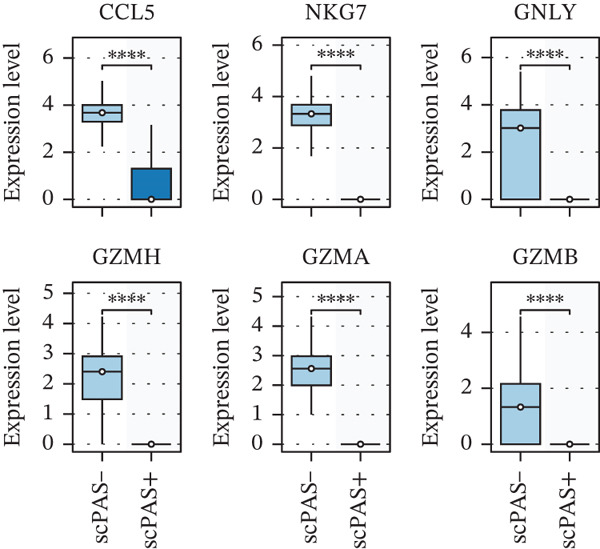
(b)

**Figure:**
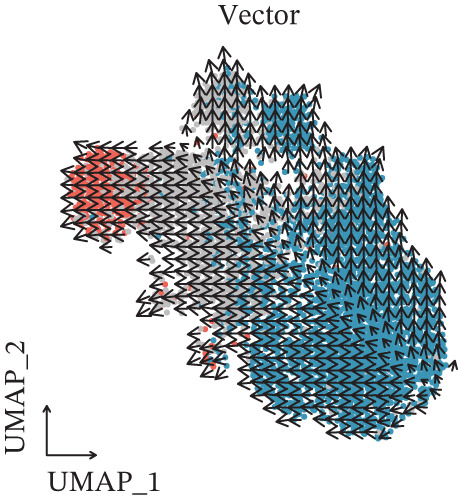
(c)

**Figure:**
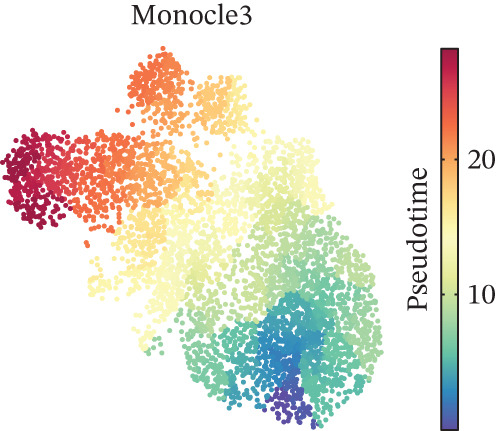
(d)

**Figure:**
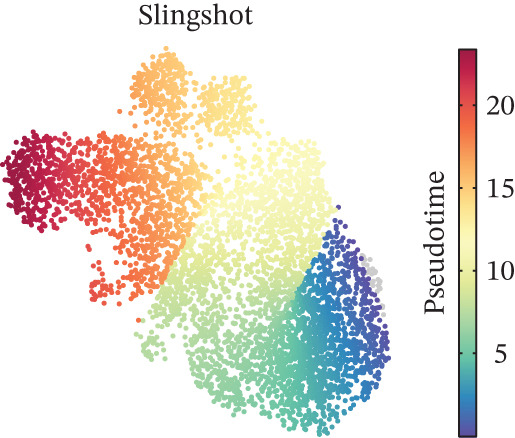
(e)

**Figure:**
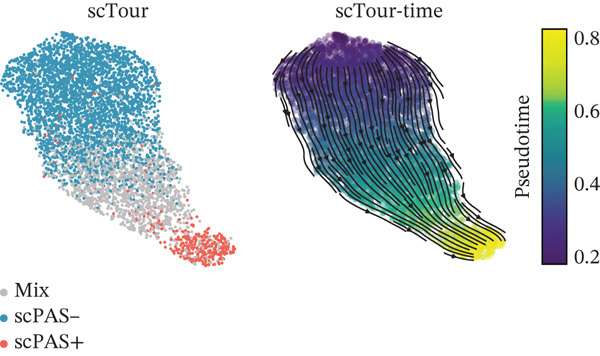
(f)

**Figure:**
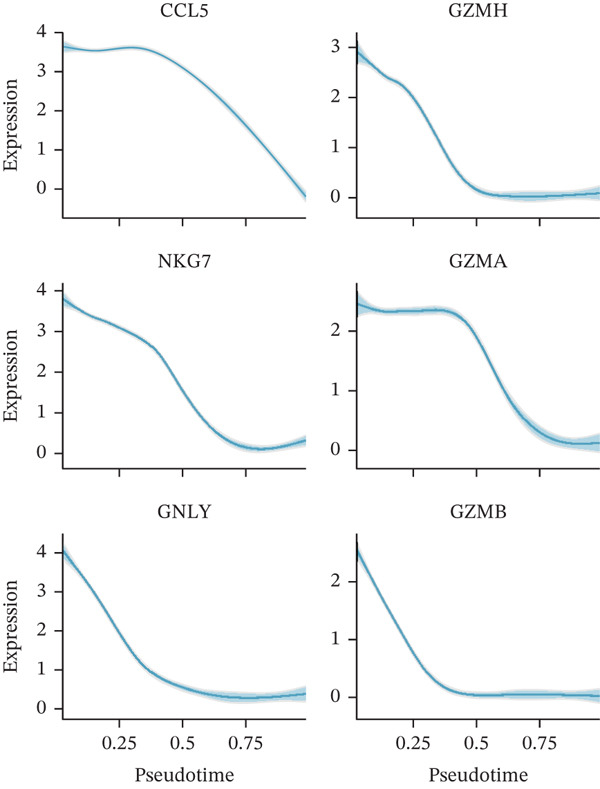
(g)

**Figure:**
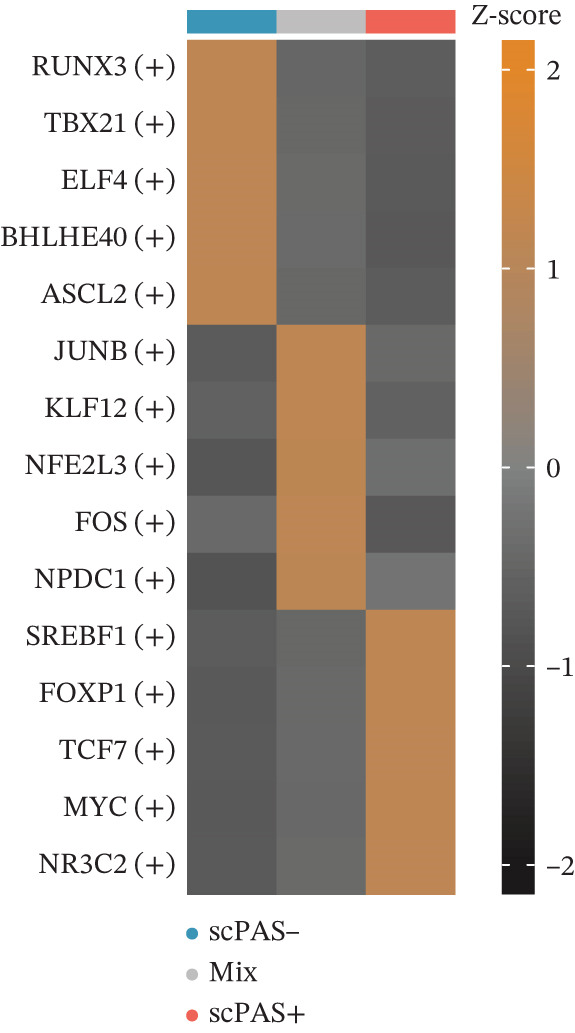
(h)

**Figure:**
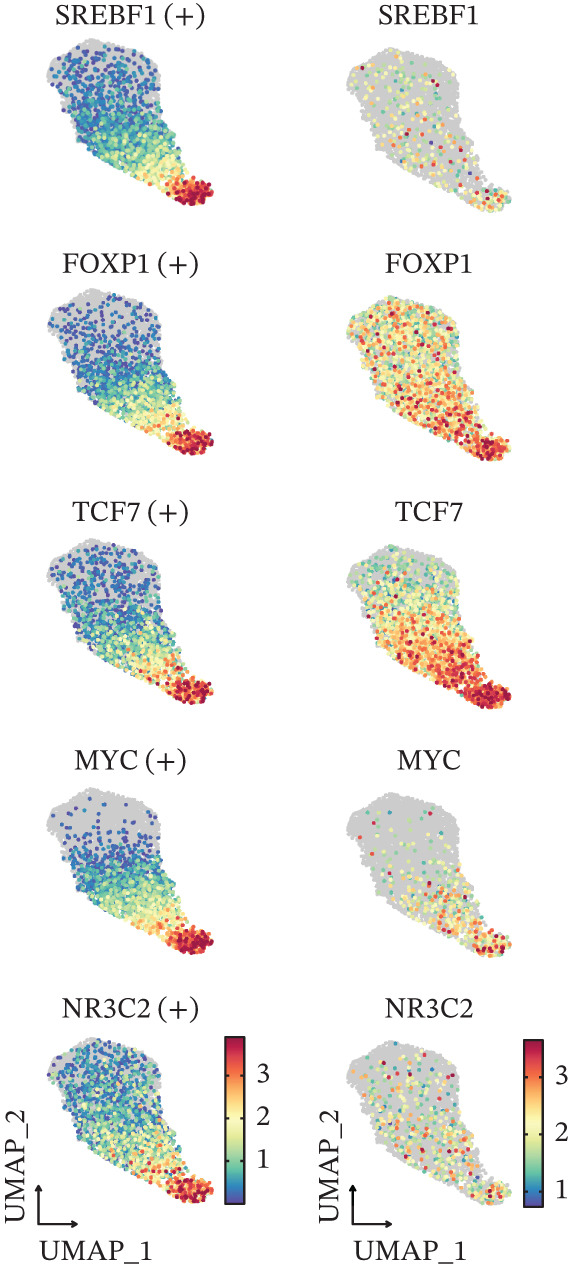
(i)

**Figure:**
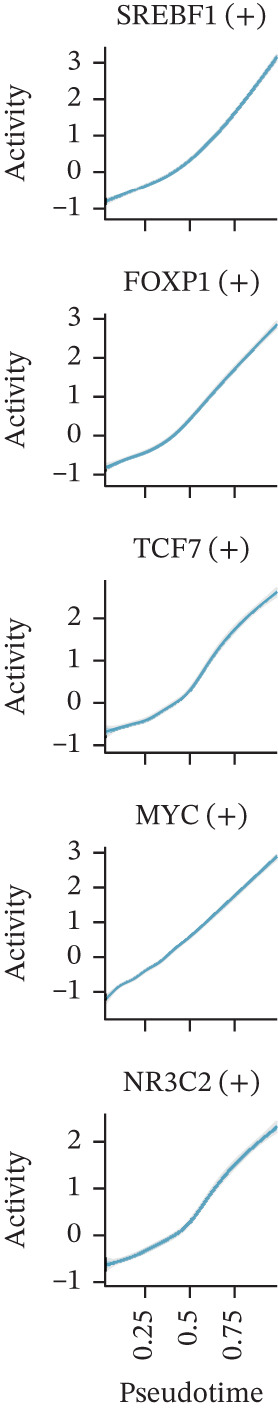
(j)

To delineate the developmental trajectory of CD8^+^ T cells, we performed pseudotime analysis. VeloCyto results (Figure 2c) depicted a trajectory from scPAS^−^ cells through mixed cells to scPAS^+^ cells, revealing a gradual transition from functionally active T cells to an exhausted phenotype. To confirm the robustness of this trajectory, we conducted orthogonal validation using three independent algorithms: Monocle3 (Figure 2d), Slingshot (Figure 2e), and scTour (Figure 2f). Remarkably, all methods yielded consistent results. The expression of CCL5, NKG7, GNLY, GZMH, GZMA, and GZMB progressively declined along the differentiation trajectory (Figure 2g).

Given that cellular phenotypes are often orchestrated by master transcription factors (TFs), we performed SCENIC analysis to quantify TF activity (Figure 2h). Our analysis revealed that SREBF1, FOXP1, TCF7, MYC, and NR3C2 exhibited elevated activity in scPAS^+^ cells (Figure 2i), with their activities showing significant positive correlation with pseudotime (Figure 2j). Conversely, RUNX3, TBX21, ELF4, BHLHE40, and ACSL2 displayed enhanced activity in scPAS^−^ cells (Figure 2h). In summary, our results establish CD8^+^ T cell dysfunction as a defining characteristic of ICH patients and provide preliminary identification of key transcriptional drivers underlying this dysfunctional phenotype.

### 3.3. scPAS^+^ Cells Exhibit Impaired Cell–Cell Communication Within the Immune Microenvironment

Intercellular communication among immune cells is essential for maintaining immune homeostasis and coordinating immune responses. The effector function of CD8^+^ T cells depends not only on their intrinsic properties but also on regulatory signals from other cell types within the immune microenvironment. Given the pronounced functional dysfunction observed in scPAS^+^ cells, we investigated whether their cell–cell communication patterns were altered in the peripheral blood immune microenvironment. Using the CellChat algorithm, we systematically analyzed the ligand–receptor interaction networks between scPAS^+^/scPAS^−^ cells and other immune cell populations, including monocytes, B cells, NK cells, and neutrophils. When scPAS^+^ and scPAS^−^ cells served as signal‐receiving cells (Figure 3a), we observed substantial signaling remodeling. Specifically, the CD86–CD28 costimulatory signal between monocytes and scPAS^+^ cells was enhanced. This pathway canonically provides the secondary signal for T cell activation; however, in the context of cellular dysfunction, this enhancement may reflect a compensatory yet ineffective attempt at activation. Additionally, the FCER2A−(ITGAX+ITGB2) signal between B cells and scPAS^+^ cells was upregulated, suggesting that B cells may attempt to establish contact with dysfunctional CD8^+^ T cells through integrin‐mediated adhesion. More strikingly, numerous critical immunoregulatory signals were abolished or markedly attenuated in scPAS^+^ cells. The CLEC2B−KLRB1 signal, which plays an important regulatory role in NK and T cell activation, was lost between scPAS^+^ cells and multiple immune cell types. Similarly, the CLEC2D−KLRB1 signal between scPAS^+^ cells and B cells, monocytes, and NK cells was significantly downregulated; this C‐type lectin receptor–mediated signaling is crucial for lymphocyte immune surveillance function. The loss of NAMPT−(ITGA5+ITGB1) signaling may impair extracellular matrix–mediated T cell survival signals. Notably, prostaglandin E2 (PGE2)–related signaling pathways, including PGE2−PTGES3−PTGER2 and PGE2−PTGES3−PTGER4, were markedly diminished in scPAS^+^ cells. Given the complex role of PGE2 in regulating T cell differentiation and function, these alterations may further exacerbate CD8^+^ T cell dysfunction. The absence of PPIA−BSG signaling suggests impaired cellular migration and tissue localization capacity.

Figure 3scPAS^+^ cells exhibit impaired cell–cell communication within the immune microenvironment. (a) Cell–cell communication analysis with scPAS^+^ and scPAS^−^ cells as receptor cells. (b) Cell–cell communication analysis with scPAS^+^ and scPAS^−^ cells as ligand cells.

**Figure:**
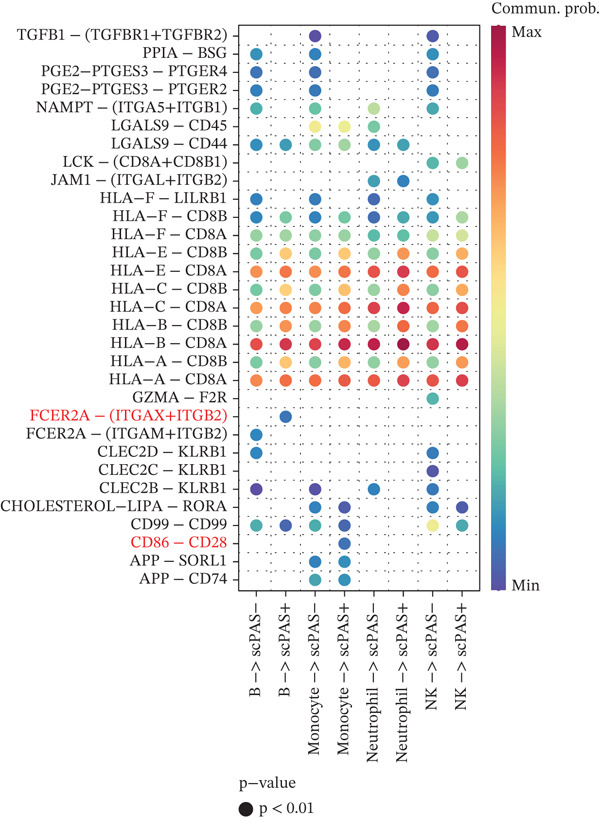
(a)

**Figure:**
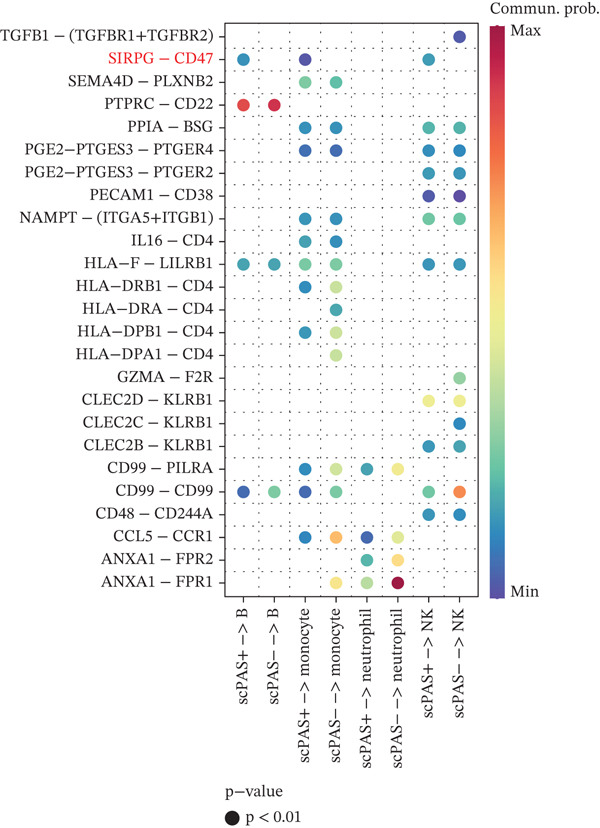
(b)

When scPAS^+^ and scPAS^−^ cells functioned as signal‐sending cells (Figure 3b), the signaling landscape was similarly restructured. The SIRPG−CD47 signal between scPAS^+^ cells and B cells, monocytes, and NK cells was enhanced. This upregulation of the “don′t eat me” signal may represent an adaptive mechanism whereby scPAS^+^ cells maintain survival by inhibiting phagocytosis. However, multiple effector‐related signals were substantially downregulated. The attenuation of ANXA1−FPR1 and ANXA1−FPR2 signaling suggests disruption of anti‐inflammatory regulatory networks between scPAS^+^ cells and myeloid cells. Consistent with our observation of CCL5 downregulation in Result 2, the CCL5−CCR1 chemotactic signal between scPAS^+^ cells and neutrophils/monocytes was markedly weakened, potentially affecting immune cell recruitment and cooperation. The loss of CD99−PILRA and CD99−CD99 signals may further compromise physical cell–cell contacts and signal transduction. Of particular note, the CLEC2C−KLRB1 and GZMA−F2R signals with NK cells were also significantly downregulated, with the latter corroborating our earlier findings of decreased granzyme expression and further confirming the comprehensive loss of cytotoxic function in scPAS^+^ cells.

In conclusion, our cell–cell communication analysis revealed extensive remodeling of the communication network involving scPAS^+^ cells within the immune microenvironment, characterized by the reduction or loss of most immunoregulatory signals. This communication impairment may be both a consequence and a contributing factor to CD8^+^ T cell dysfunction, potentially creating a vicious cycle. These findings provide novel insights into the molecular mechanisms underlying peripheral immune dysfunction in ICH patients and suggest that therapeutic strategies aimed at restoring normal communication between CD8^+^ T cells and other immune cells may represent a promising intervention approach.

### 3.4. Multialgorithm Machine Learning Integration Identifies CD8^+^ T Cell–Derived SELL as a Robust Diagnostic Biomarker for ICH Patients

Early identification of specific biomarkers for ICH patients is of paramount importance for timely clinical diagnosis and therapeutic decision‐making. Building upon the scPAS^+^ dysfunctional CD8^+^ T cells identified in our preceding analyses, we sought to discover biomarkers with clinical translational potential. To this end, we systematically integrated single‐cell and bulk transcriptomic data to identify key molecules exhibiting consistent alterations at both cellular and tissue levels. Specifically, we employed a bidirectional intersection strategy: We intersected genes significantly upregulated in scPAS^+^ cells with those upregulated in ICH patients from bulk transcriptomic Cohort 1, while simultaneously intersecting genes downregulated in scPAS^+^ cells with those downregulated in Cohort 1. This approach yielded 60 key genes showing concordant changes at both single‐cell and bulk levels (Figure 4a). GO enrichment analysis revealed that these genes were significantly enriched in pathways including lymphocyte‐mediated immunity, T cell–mediated immunity, cell killing, regulation of T cell–mediated immunity, regulation of T cell activation, and leukocyte cell–cell adhesion (Figure 4b), indicating their close association with CD8^+^ T cell functional regulation and further validating the reliability of our screening strategy.

Figure 4Multialgorithm machine learning integration identifies CD8^+^ T cell–derived SELL as a robust diagnostic biomarker for ICH patients. (a) Intersection of differentially expressed genes identified from scPAS^+^ cells and bulk cohort analyses. (b) Functional enrichment analysis of the overlapping genes. (c) Feature gene selection using LASSO regression. (d) Optimization of the ntree parameter during random forest feature selection. (e) Top 20 genes ranked by importance from random forest analysis. (f) Feature importance ranking from XGBoost analysis. (g) Sixteen genes selected by the SVM algorithm. (h) Genes identified by the Boruta algorithm. (i) Intersection of five machine learning algorithms identifying SELL as a consensus gene for ICH. (j) Significantly elevated expression of SELL in ICH patients in Cohort 1 and Cohort 2. (k) ROC curves of SELL for diagnosing ICH in Cohort 1 and Cohort 2.

**Figure:**
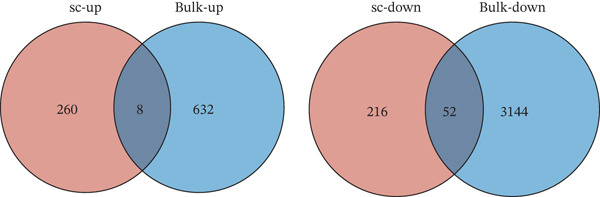
(a)

**Figure:**
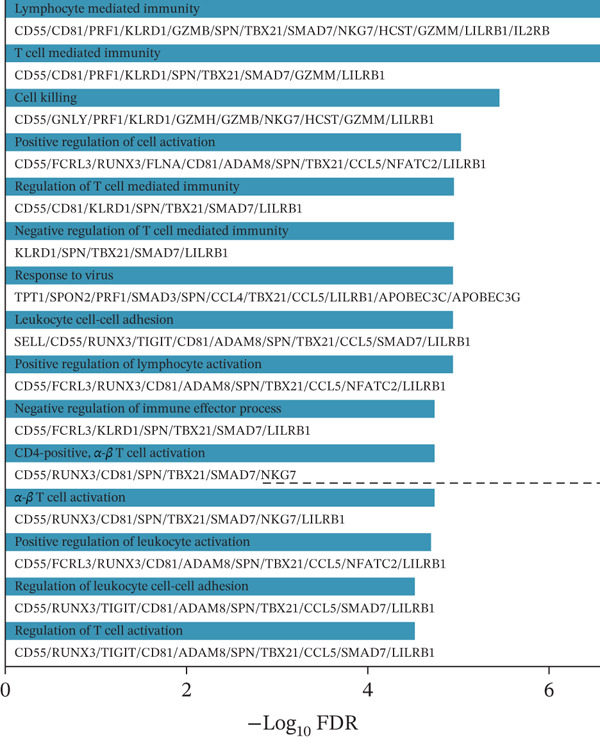
(b)

**Figure:**
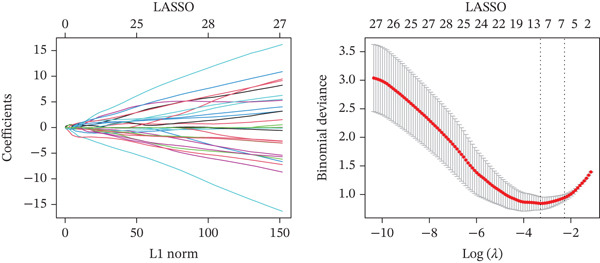
(c)

**Figure:**
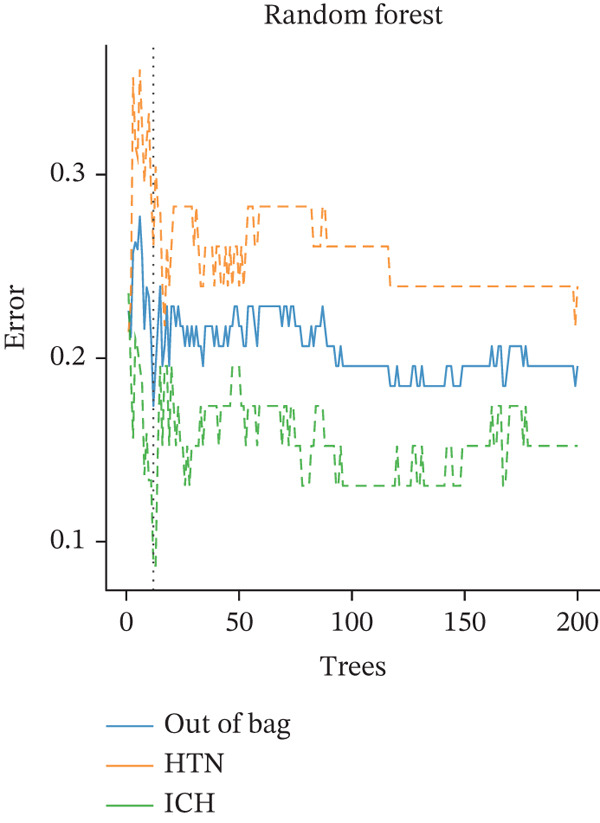
(d)

**Figure:**
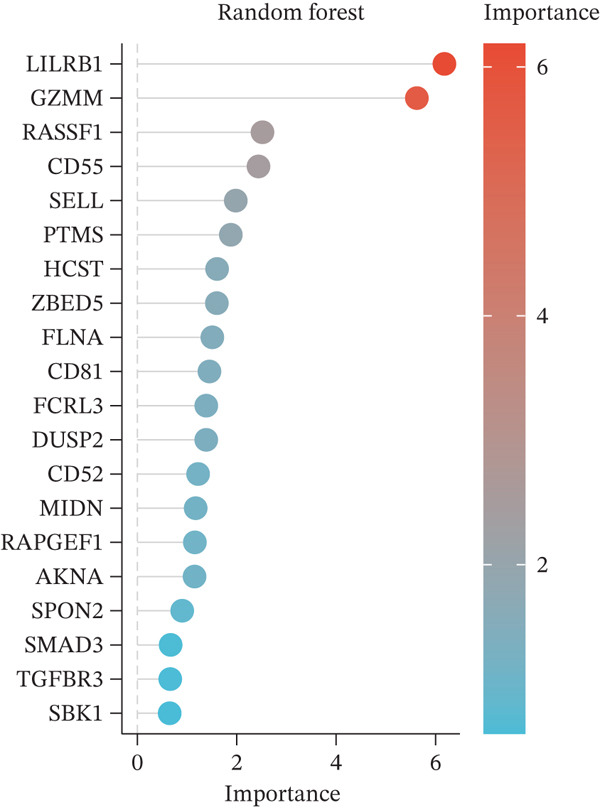
(e)

**Figure:**
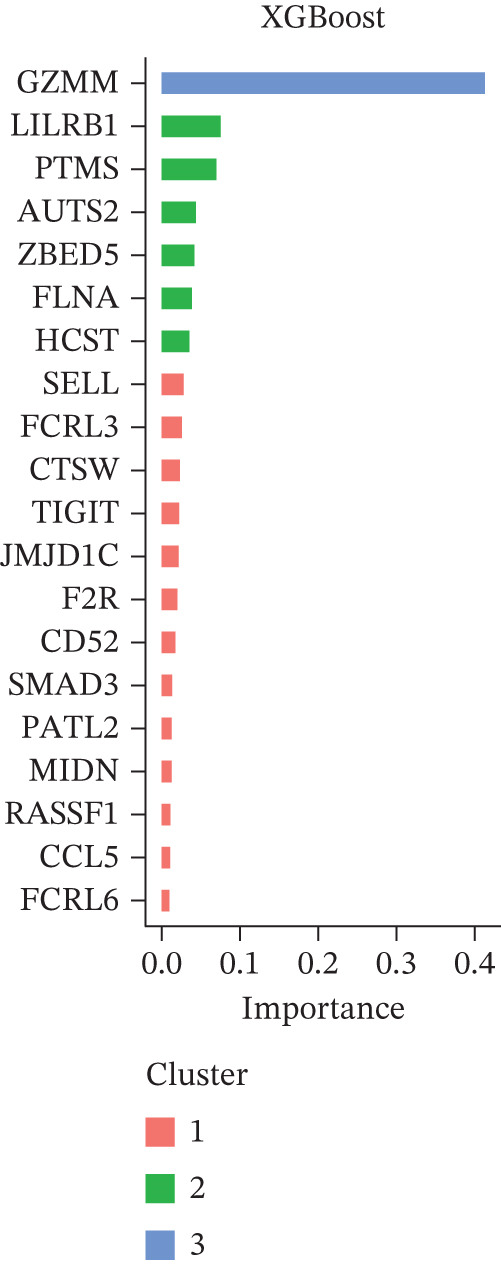
(f)

**Figure:**
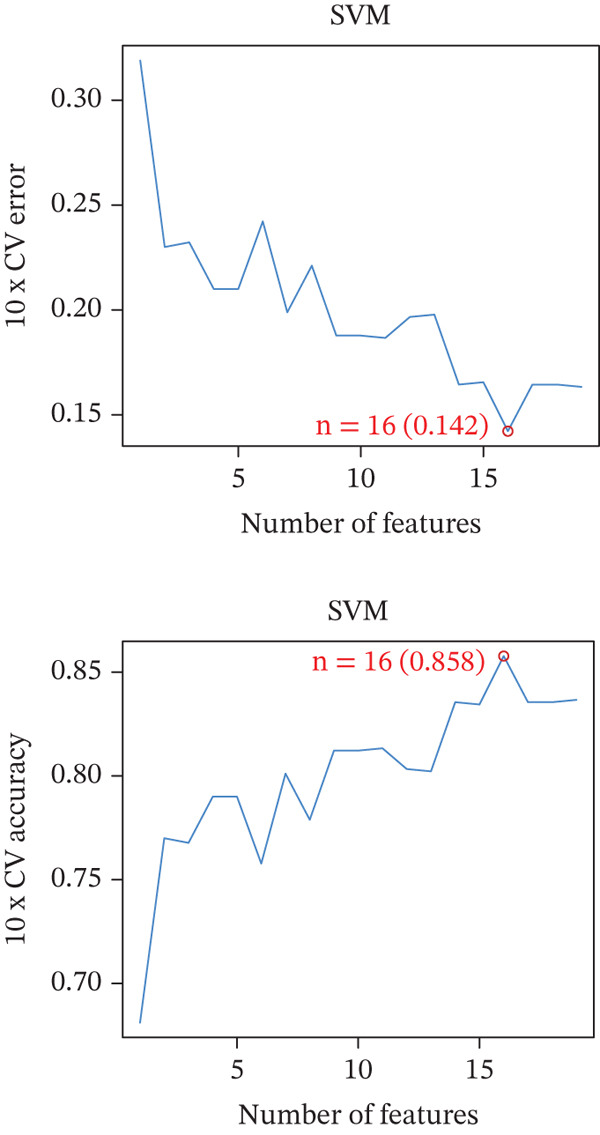
(g)

**Figure:**
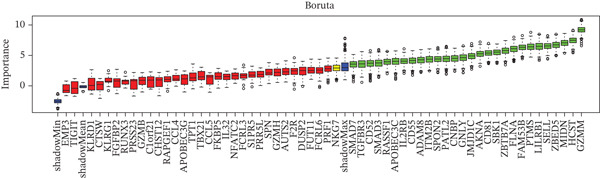
(h)

**Figure:**
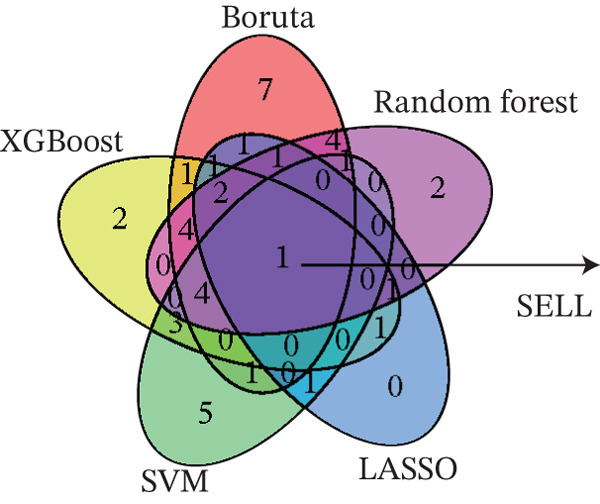
(i)

**Figure:**
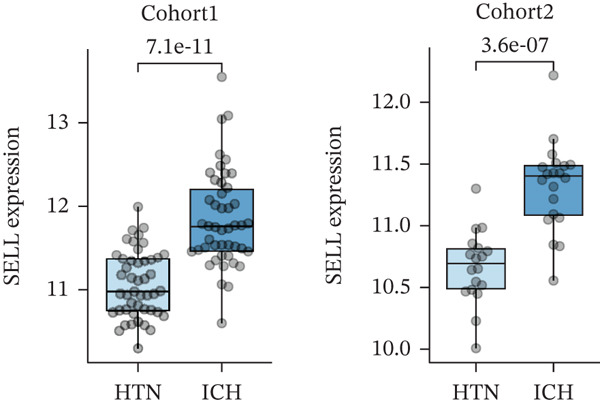
(j)

**Figure:**
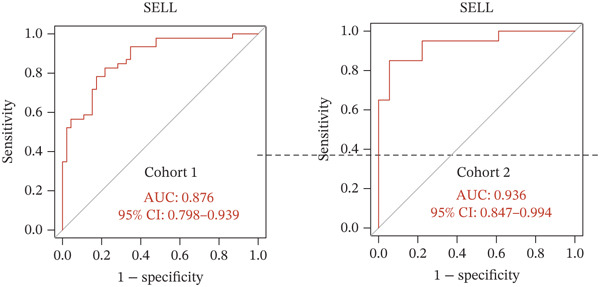
(k)

To precisely identify the most diagnostically valuable core biomarker from these 60 candidate genes, we integrated five complementary machine learning feature selection algorithms: LASSO regression, random forest, extreme gradient boosting (XGBoost), support vector machine (SVM), and Boruta. The results from each algorithm were as follows: LASSO regression, through L1 regularization penalty, identified nine feature genes (Figure 4c); random forest selected the Top 20 genes based on feature importance scores (Figure 4d,e); XGBoost identified 20 feature genes with high predictive contribution (Figure 4f); SVM, employing recursive feature elimination, achieved optimal classification accuracy with 16 feature genes (Figure 4g); and Boruta, as a wrapper‐based feature selection method, confirmed 28 significant feature genes (Figure 4h). Remarkably, despite the distinct underlying principles and emphases of each algorithm, the intersection of results from all five machine learning approaches converged on a single consensus gene—SELL (Figure 4i). SELL encodes L‐selectin (CD62L), a cell adhesion molecule expressed on the lymphocyte surface that participates in lymphocyte homing and migration. A portion of SELL can also exist in a secreted form in the extracellular space. We validated the expression changes and diagnostic performance of SELL in independent cohorts. In bulk transcriptomic Cohort 1, SELL expression levels were significantly elevated in the peripheral blood of ICH patients compared to HTN controls (Figure 4j). Importantly, this finding was consistently replicated in the validation cohort (Cohort 2) (Figure 4j), demonstrating the robustness of this observation. ROC curve analysis further evaluated the diagnostic performance of SELL as an ICH biomarker: In Cohort 1, the area under the curve (AUC) for SELL reached 0.876 (Figure 4k); in the independent validation Cohort 2, the AUC was even higher at 0.936 (Figure 4k), indicating excellent and stable diagnostic performance.

In summary, through integrating single‐cell and bulk transcriptomic multiomics analyses combined with cross‐validation using multiple machine learning algorithms, we identified and validated CD8^+^ T cell–derived SELL as a robust diagnostic biomarker for ICH patients. This discovery not only provides a potential molecular tool for early ICH diagnosis but also further supports the critical role of CD8^+^ T cell dysfunction in the pathogenesis of ICH.

### 3.5. Targeting CD62L With Rutin Attenuates Hypertensive ICH

To investigate the role of CD62L in ICH, we established mouse models of HTN and ICH (Figure 5a). First, we analyzed the expression of T cell cytotoxicity–associated genes in peripheral blood. The results revealed a significant suppression of effector genes, including Ccl5, Gnly, Gzma, Gzmb, Gzmh, and Nkg7, in the ICH group compared with HTN controls. Notably, Sell mRNA expression was upregulated, consistent with its putative role in mitigating ICH‐induced damage (Figure 5b). Furthermore, ELISA analysis demonstrated that CD62L protein levels in peripheral blood were significantly elevated in the ICH group (Figure 5c). To determine whether CD62L expression in CD8^+^ T cells mirrors these changes observed in blood samples, we isolated CD8^+^ T cells using FACS and measured CD62L expression levels. Western blot analysis revealed that CD62L expression was significantly increased in CD8^+^ T cells from ICH mice compared with HTN controls (Figure 5d). Immunofluorescence staining further demonstrated that CD62L was predominantly expressed in CD8^+^ T cells within the brain, with markedly enhanced fluorescence intensity following ICH (Figure 5e). Flow cytometry confirmed a significant increase in the proportion of CD62L^+^ cells among CD8^+^ T cells (Figure 5f). Collectively, these findings indicate that CD62L derived from CD8^+^ T cells may serve as a specific biomarker for ICH.

Figure 5Targeting CD62L with rutin attenuates hypertensive intracerebral hemorrhage. (a) Schematic illustration of mouse models for hypertension (HTN) versus induced intracerebral hemorrhage (ICH). (b) Quantitative PCR analysis of Ccl5, Gnly, Gzma, Gzmb, Gzmh, Nkg7, and Sell expression in peripheral blood. *n* = 6 per group. (c) Serum CD62L levels measured by ELISA. *n* = 5 per group. (d) Western blot analysis of CD62L expression in CD8^+^ T cells from HTN and ICH groups. *n* = 6 per group. (e) Representative immunofluorescence images of CD62L expression in CD8^+^ T cells within cerebral vessels. Scale bar = 20 * μ*m. (f) Flow cytometry quantification of CD62L^+^ cells among CD8^+^ T cells in peripheral blood from the HTN and ICH groups. *n* = 6 per group. (g) Identification of high‐affinity small molecules targeting CD62L through molecular docking analysis. (h) Molecular docking analysis showing the interaction between rutin and CD62L protein, including binding sites and interaction patterns. (i) Western blot analysis of CD62L expression in CD8^+^ T cells from the DMSO+AL and rutin+AL groups. AL, AngII plus L‐NAME. *n* = 6 per group. (j) Flow cytometry quantification of CD62L^+^ cells among CD8^+^ T cells in peripheral blood from the DMSO+AL and rutin+AL groups. *n* = 6 per group. (k) Representative immunofluorescence images of CD62L expression in CD8^+^ T cells within cerebral vessels following treatment. Scale bar = 20 * μ*m.

**Figure:**
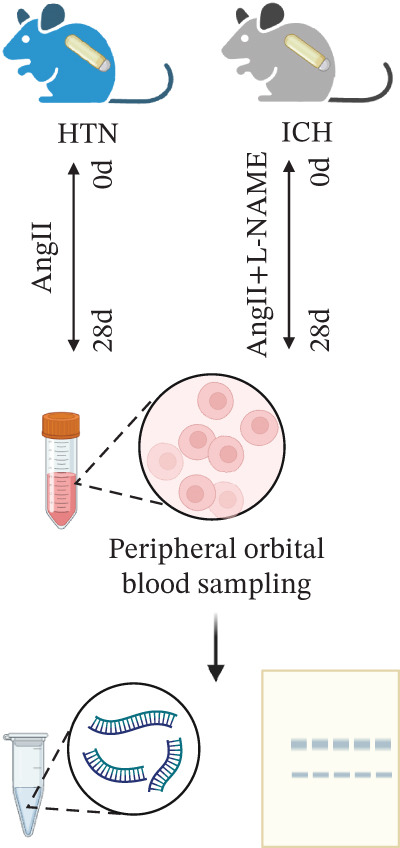
(a)

**Figure:**
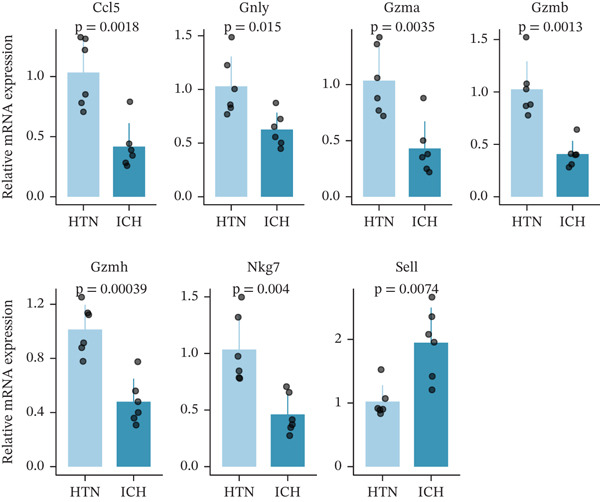
(b)

**Figure:**
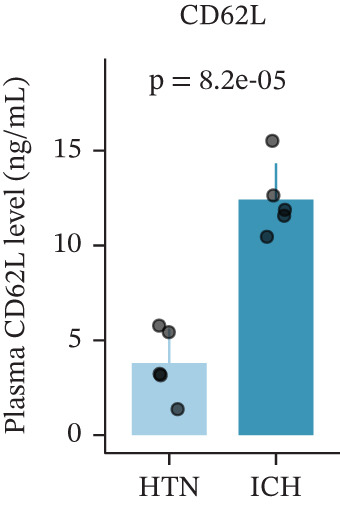
(c)

**Figure:**
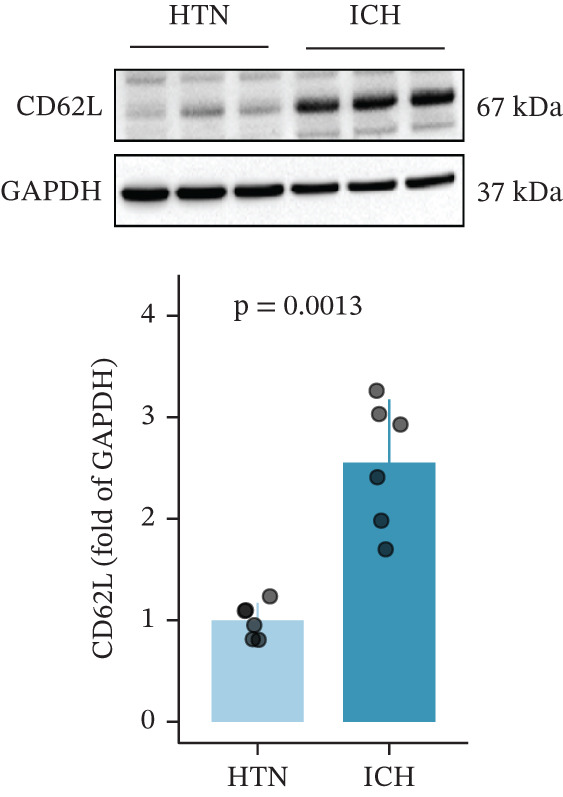
(d)

**Figure:**
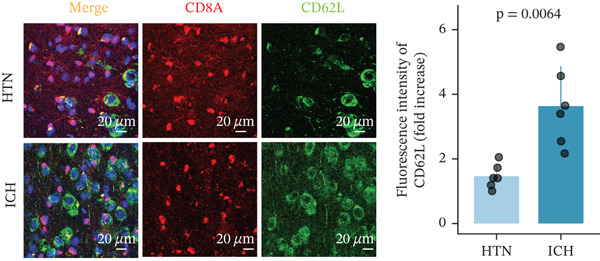
(e)

**Figure:**
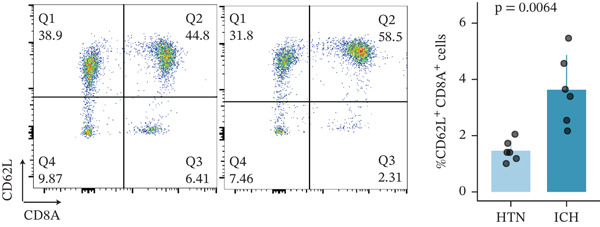
(f)

**Figure:**
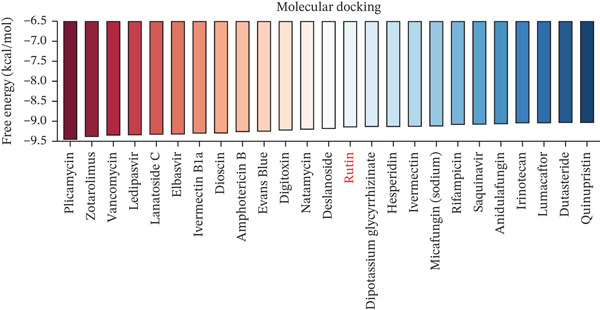
(g)

**Figure:**
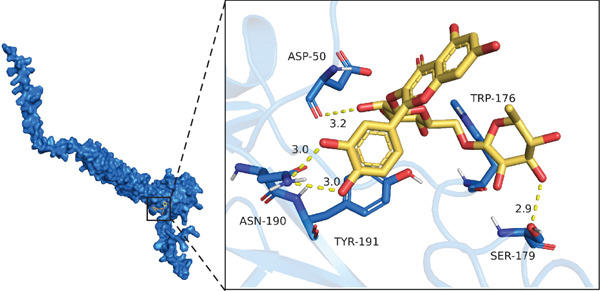
(h)

**Figure:**
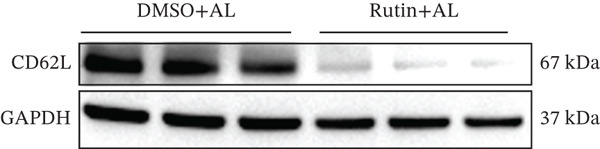
(i)

**Figure:**
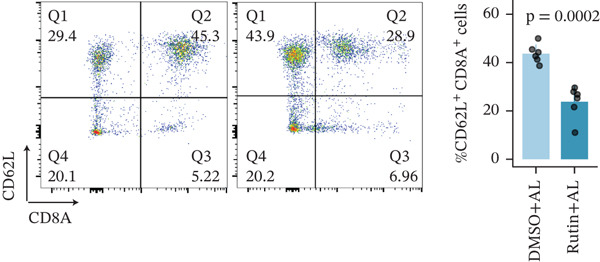
(j)

**Figure:**
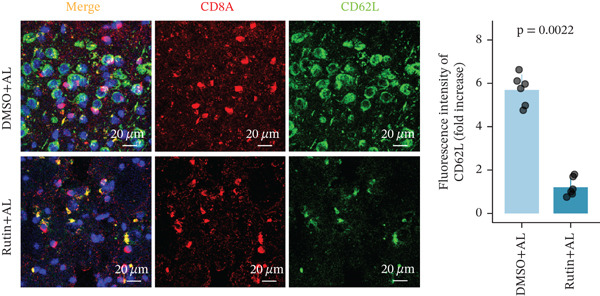
(k)

To identify potential therapeutic agents targeting CD62L, we employed molecular docking to calculate the binding affinity between CD62L and 2987 FDA‐approved small‐molecule compounds. This analysis identified 25 compounds with binding affinities below −8 kcal/mol (Figure 5g). Among these candidates, we focused on rutin due to its well‐documented antioxidant and neuroprotective properties. Molecular docking revealed that rutin forms stable interactions with CD62L through hydrogen bonds involving ASN‐190, TYR‐191, ASP‐50, TRP‐176, and SER‐179 residues (Figure 5h). To validate whether rutin exerts its effects on ICH through CD62L binding, we conducted in vivo experiments. Following 42 days of rutin administration, Western blot analysis demonstrated decreased CD62L expression in CD8^+^ T cells (Figure 5i). Flow cytometry revealed a significant reduction in the proportion of CD62L^+^ CD8^+^ T cells (Figure 5j). Additionally, immunofluorescence staining confirmed a marked decrease in CD62L fluorescence intensity within CD8^+^ T cells in cerebral vessels (Figure 5k). These results collectively demonstrate that rutin exerts protective effects against ICH by targeting CD62L.

### 3.6. Rutin Protects Against Hypertensive Cerebral Hemorrhage

To elucidate the protective mechanisms of rutin in targeting CD62L during hypertensive cerebral hemorrhage, we established an animal model of ICH. Quantitative PCR analysis revealed that rutin treatment significantly increased the expression of cytotoxicity‐associated genes (Ccl5, Gnly, Gzma, Gzmb, Gzmh, and Nkg7) while markedly downregulating Sell expression (Figure 6a). Importantly, the incidence of ICH was lower in the rutin treatment group compared with the DMSO‐treated controls (Figure 6b). To quantify hemorrhage burden, we performed T2∗‐weighted MRI to delineate and measure hematoma lesion volumes. Rutin‐treated mice exhibited a marked reduction in lesion size compared with controls. Spectrophotometric hemoglobin assays further confirmed that rutin treatment significantly decreased hemorrhage volume (Figure 6c). Histological analysis corroborated these findings, revealing an increased number and size of cerebral hemorrhages in DMSO‐treated mice compared with the rutin treatment group (Figure 6d). Chronic hypertensive stimulation promotes T cell exhaustion, which activates vascular and neuroinflammatory responses contributing to cerebral hemorrhage. Consistent with this inflammatory milieu, microglial activation—defined by altered morphology and Iba‐1^+^ soma diameter > 7.5 * μ*m—was prominent in the DMSO treatment group, indicating concomitant blood–brain barrier impairment and compromised cerebral microcirculation. Notably, rutin treatment significantly attenuated microglial activation and the associated inflammatory response (Figure 6e). Vascular smooth muscle cells (VSMCs) in cerebral small arteries and arterioles, which are essential for extracellular matrix production and vessel wall stability, exhibited significantly reduced apoptosis following rutin treatment (Figure 6f). DHE staining demonstrated that rutin treatment markedly reduced oxidative stress levels (Figure 6g). Furthermore, ELISA measurements revealed that rutin treatment significantly decreased circulating levels of the proinflammatory cytokines IL‐6 and TNF‐*α* (Figure 6h). Collectively, these results establish rutin as a protective small‐molecule agent that attenuates hypertensive ICH through targeting SELL.

Figure 6Rutin protects against hypertensive cerebral hemorrhage. (a) Quantitative PCR analysis of Ccl5, Gnly, Gzma, Gzmb, Gzmh, Nkg7, and Sell expression in peripheral blood. *n* = 6 per group. (b) Kaplan–Meier survival curve showing the incidence of stroke signs in each group. *n* = 20 per group. Log‐rank (Mantel–Cox) test. (c) Representative dorsal and sagittal brain images showing ICH across groups. T2∗‐weighted gradient‐echo MRI scans performed at Day 5 postimplantation and upon stroke onset were used to quantify lesion size and number. *n* = 6 per group. (d) Representative H&E‐stained images depicting ICH in the cortex, striatum, and hippocampus (scale bar = 200 * μ*m). Quantification of hemorrhage size distribution comparing the DMSO+AL and rutin+AL groups. *n* = 6 per group. (e) Representative immunofluorescence images and quantification of Iba‐1^+^ microglia demonstrating activated microglia surrounding cerebral vessels. *n* = 6 per group. (f) Representative images and quantitative assessment of apoptosis in *α*‐SMA vascular smooth muscle cells at Day 5 posttreatment in AL‐treated mice. *n* = 6 per group. (g) Reactive oxygen species (ROS) levels assessed by dihydroethidium (DHE) fluorescence staining, with red signal intensity indicating relative ROS levels. *n* = 6 per group. (h) Serum levels of proinflammatory cytokines IL‐6 (left) and TNF‐*α* (right) measured by ELISA. *n* = 6 per group.

**Figure:**
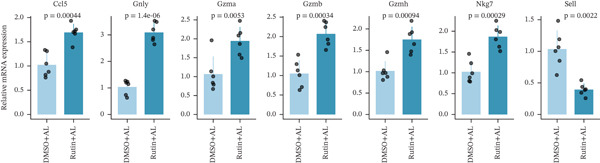
(a)

**Figure:**
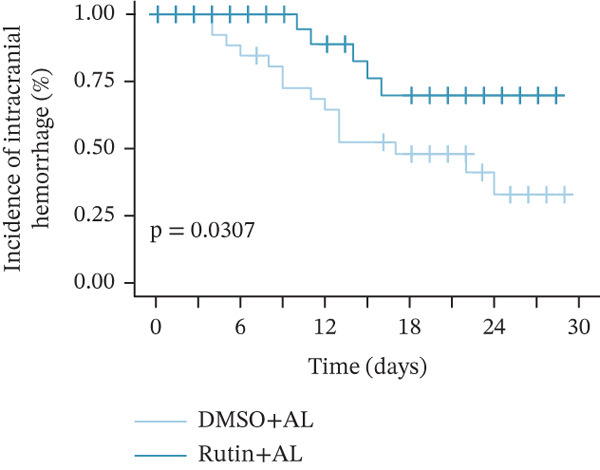
(b)

**Figure:**
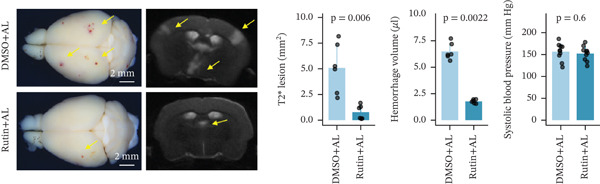
(c)

**Figure:**
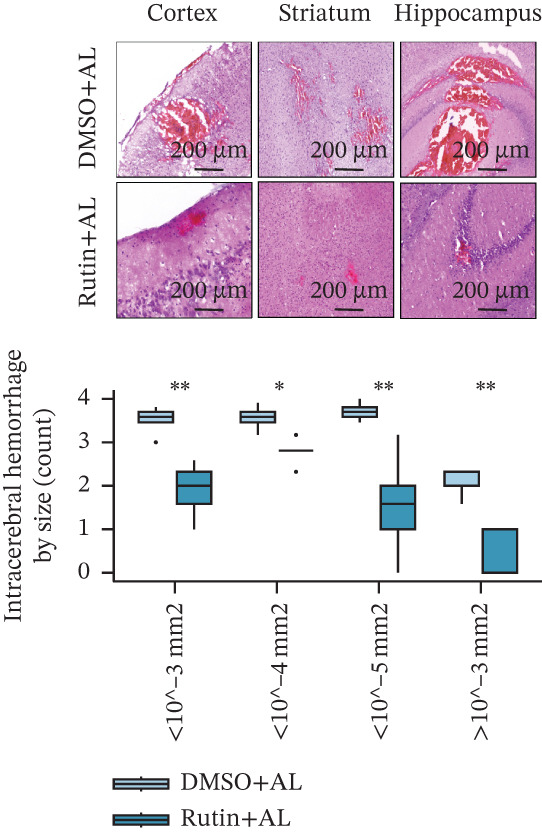
(d)

**Figure:**
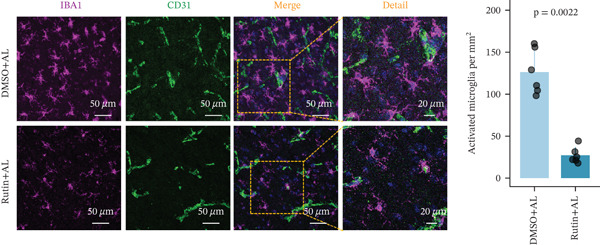
(e)

**Figure:**
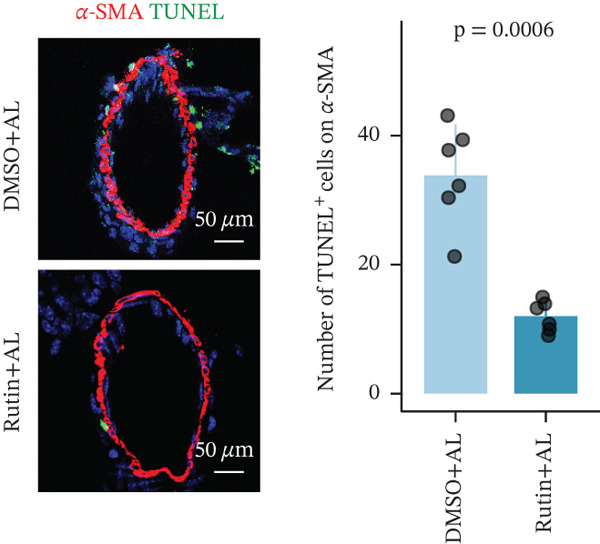
(f)

**Figure:**
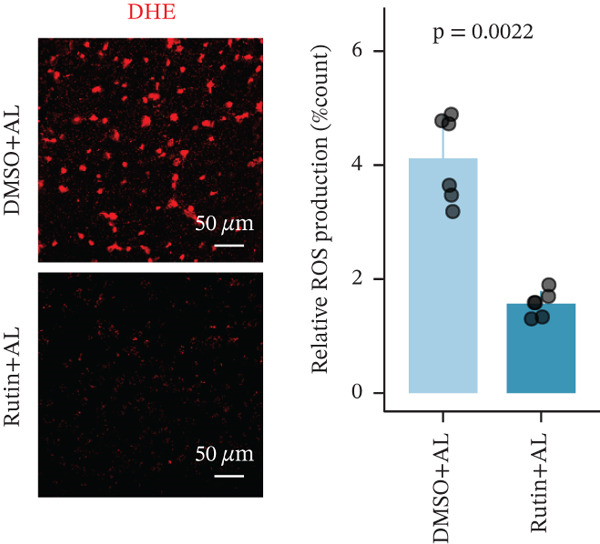
(g)

**Figure:**
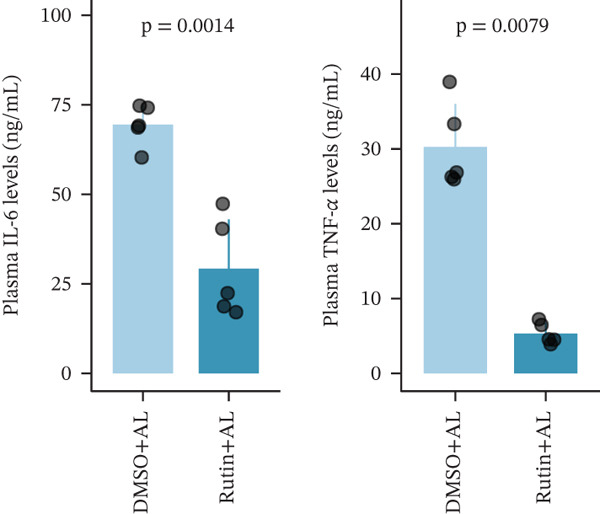
(h)

## 4. Discussion

The present study establishes CD8^+^ T cell dysfunction as a defining immunological characteristic of ICH patients and identifies CD62L as a robust diagnostic biomarker with exceptional discriminative performance. Through systematic integration of bulk and single‐cell transcriptomic analyses, we delineated a phenotypically distinct SELL^high^ CD8^+^ T cell subpopulation exhibiting comprehensive effector impairment, designated as scPAS^+^ cells. Moreover, our pharmacological investigations demonstrated that rutin effectively attenuates hypertensive ICH through targeted modulation of SELL expression, establishing this flavonoid compound as a promising therapeutic candidate.

Our multiomics approach revealed pronounced suppression of T cell receptor signaling and cytotoxic effector pathways in ICH patient peripheral blood, consistent with previous observations of systemic immunodepression following acute cerebrovascular events [39, 40]. Strikingly, the scPAS algorithm enabled precise identification of disease‐associated CD8^+^ T cell populations through integrative analysis of single‐cell and bulk transcriptomic signatures. The scPAS^+^ cells demonstrated coordinated downregulation of cytotoxicity‐associated genes, including GZMA, GZMB, GZMH, GNLY, NKG7, and CCL5, indicating a fundamental impairment in cell‐mediated immune functions. This effector deficiency phenotype aligns with emerging conceptualizations of T cell exhaustion, a dysfunctional state originally characterized in chronic viral infections and malignancies [41, 42].

Pseudotime trajectory analyses utilizing multiple complementary algorithms (VeloCyto, Monocle3, Slingshot, and scTour) converged on a consistent differentiation trajectory from functional scPAS^−^ cells through intermediate populations toward dysfunctional scPAS^+^ cells. The progressive decline in granzyme and perforin expression along this trajectory mirrors the gradual exhaustion program observed in tumor‐infiltrating lymphocytes and chronic infection models [43, 44]. TF activity profiling identified FOXP1, TCF7, and MYC as potential drivers of the scPAS^+^ phenotype, which have established roles in regulating T cell quiescence and memory formation [45, 46]. Conversely, effector‐associated TFs RUNX3 and TBX21 exhibited diminished activity in scPAS^+^ cells, corroborating the functional impairment phenotype.

The cell–cell communication analyses revealed extensive remodeling of intercellular signaling networks involving scPAS^+^ cells within the peripheral immune microenvironment. Notably, critical immunoregulatory pathways, including CLEC2B–KLRB1 and CLEC2D–KLRB1, were substantially attenuated, potentially compromising coordinated immune surveillance functions. The concurrent upregulation of SIRPG–CD47 signaling suggests a compensatory mechanism whereby dysfunctional T cells resist phagocytic clearance [47]. These communication deficits may establish a self‐perpetuating cycle wherein impaired intercellular crosstalk further exacerbates CD8^+^ T cell dysfunction. Therapeutic strategies aimed at restoring normal immune cell communication networks may therefore represent a promising intervention paradigm for ICH.

The identification of SELL as a consensus biomarker through the intersection of five independent machine learning algorithms (LASSO, random forest, XGBoost, SVM, and Boruta) substantially strengthens the robustness and reliability of this finding. Machine learning–based feature selection has emerged as a powerful approach for biomarker discovery in complex diseases, enabling objective identification of diagnostically valuable molecular signatures from high‐dimensional datasets [48, 49]. The remarkable consistency across methodologically diverse algorithms, each employing distinct underlying principles, provides compelling evidence for SELL′s genuine association with ICH pathogenesis rather than representing a statistical artifact. The excellent diagnostic performance demonstrated in both discovery (AUC = 0.876) and independent validation (AUC = 0.936) cohorts further substantiates the clinical utility of SELL as a peripheral blood biomarker for ICH identification.

From a mechanistic perspective, SELL overexpression in scPAS^+^ cells may reflect impaired T cell activation dynamics. Under physiological conditions, CD62L undergoes rapid proteolytic shedding upon T cell receptor engagement, facilitating transition from naive/central memory to effector phenotypes [22, 50]. The retention of elevated SELL expression in disease‐associated CD8^+^ T cells may therefore indicate aberrant activation signaling or enhanced lymph node homing that diverts cells from effector differentiation. Previous investigations have documented that transgenic mice expressing noncleavable CD62L demonstrate compromised viral clearance capacity, suggesting that SELL retention functionally impairs T cell–mediated immunity [51]. Our findings extend these observations to the context of hypertensive ICH, positioning SELL as both a biomarker and potential therapeutic target.

The therapeutic efficacy of rutin in our murine ICH models provides proof‐of‐concept validation for CD62L‐targeted intervention strategies. Rutin administration effectively reduced CD62L expression in CD8^+^ T cells, restored cytotoxic gene expression profiles, and attenuated hemorrhage incidence and volume. These protective effects were accompanied by reduced microglial activation, decreased VSMC apoptosis, and diminished oxidative stress burden. The neuroprotective mechanisms of rutin are multifaceted, encompassing direct antioxidant activity through radical scavenging, anti‐inflammatory effects via NF‐*κ*B pathway suppression, and blood–brain barrier preservation through matrix metalloproteinase inhibition [28, 52, 53]. Our molecular docking analyses identified specific hydrogen bonding interactions between rutin and CD62L residues (ASN‐190, TYR‐191, ASP‐50, TRP‐176, and SER‐179), providing structural rationale for the observed modulation of SELL expression.

The present findings harbor important translational implications. First, peripheral blood SELL quantification offers a minimally invasive approach for ICH risk stratification that could complement existing neuroimaging modalities. Second, the identification of an effector‐deficient CD8^+^ T cell phenotype suggests that immunomodulatory interventions targeting T cell dysfunction may ameliorate posthemorrhagic brain injury. Third, rutin represents a readily available, well‐tolerated natural compound with established safety profiles that warrants clinical investigation as adjunctive therapy for hypertensive ICH. The convergence of biomarker discovery and therapeutic target identification within a unified analytical framework exemplifies the potential of integrative multiomics approaches for advancing precision medicine in cerebrovascular diseases.

Several limitations of this study merit acknowledgment. First, the cross‐sectional design of our patient cohorts precludes definitive establishment of causal relationships between SELL expression and ICH occurrence. Longitudinal studies tracking SELL dynamics preceding and following hemorrhagic events would strengthen causal inference. Second, our single‐cell analyses were performed on cerebral MB patients as a surrogate for early‐stage hemorrhagic pathology; validation in acute ICH cohorts would enhance clinical relevance. Third, while our murine models recapitulate key features of hypertensive ICH, species‐specific differences in immune cell biology may limit direct extrapolation to human disease. Fourth, the precise molecular mechanisms underlying rutin‐mediated SELL modulation require further elucidation through comprehensive pharmacokinetic and pharmacodynamic investigations. Fifth, a limitation of this study is the clinical heterogeneity between the MB discovery cohort and the ICH validation cohort. Although these conditions represent a pathophysiological continuum of small vessel disease, their immune profiles may differ in magnitude. Our findings likely highlight core conserved pathways, yet these results should be further validated in longitudinal, stage‐matched cohorts.

In conclusion, this study provides a comprehensive characterization of peripheral CD8^+^ T cell dysfunction in ICH pathogenesis and identifies SELL‐expressing effector‐deficient T cells as a disease‐associated subpopulation. The exceptional diagnostic performance of SELL across independent cohorts positions this molecule as a promising biomarker for clinical translation. Furthermore, the therapeutic efficacy of rutin in attenuating hypertensive ICH through SELL targeting establishes a foundation for developing immunomodulatory interventions in hemorrhagic stroke. These findings collectively advance our mechanistic understanding of neuroimmune interactions in ICH and illuminate actionable therapeutic avenues warranting further clinical investigation.

**Nomenclature**
Ang II: Angiotensin II
AL: Angiotensin II and ^*ω*^‐nitro‐L‐arginine methyl ester
ARRIVE: Animal Research: Reporting of In Vivo Experiments
BP: blood pressure
DHE: dihydroethidium
DMSO: dimethyl sulfoxide
SELL: selectin L
TNF‐*α*
: tumor necrosis factor‐alpha
WT: wild type

## Author Contributions

Jingzhou Chen initiated the project, conceptualized the study, designed the experimental framework, and acquired the funding to support this work. Yan Huang and Haochen Xu designed and implemented the software and algorithms for data analysis, performed validation experiments, and carried out the investigations. Yan Huang conducted the formal statistical and bioinformatic analyses and wrote the original draft of the manuscript. Congxia Bai, Jing Liu, and Yingying Sun provided critical reagents, cell lines, and laboratory instrumentation. Yan Huang and Haochen Xu contributed equally to this work and share first authorship.

## Funding

The authors disclosed receipt of the following financial support for the research, authorship, and/or publication of this article: This work was supported by the National Science and Technology Major Project for Non‐communicable Chronic Diseases (Grant No. 2023ZD0503201 to J.C.), the National Natural Science Foundation of China (Grant No. 82130013 to J.C.), the National High Level Hospital Clinical Research Funding (Grant No. 2025‐GSP‐GG‐27 to J.C.), and the CAMS Innovation Fund for Medical Sciences (Grant No. 2023‐I2M‐2‐003 to J.C.).

## Ethics Statement

This study complied with the Declaration of Helsinki and was approved by the ethics committee of the Fuwai Hospital (Approval No. 2024‐2458).

## Consent

All authors have provided consent for the publication of this work.

## Conflicts of Interest

The authors declare no conflicts of interest.

## Data Availability

The data that support the findings of this study are available from the corresponding author upon reasonable request.
